# Incidence, Prevalence, and Stability of Remission in Individuals With Clinical High Risk for Psychosis

**DOI:** 10.1001/jamanetworkopen.2025.25644

**Published:** 2025-08-05

**Authors:** Johanna Seitz-Holland, Grace R. Jacobs, Jenna Reinen, Daniel Mathalon, Cheryl Corcoran, Abraham Reichenberg, Mark Vangel, Robert J. Glynn, Nora Penzel, Kang-Ik K. Cho, Eduardo Castro, Anastasia Haidar, Jean M. Addington, Tina Kapur, Sylvain Bouix, Carrie E. Bearden, John M. Kane, Patrick D. McGorry, Scott W. Woods, Barnaby Nelson, René S. Kahn, Martha E. Shenton, Guillermo A. Cecchi, Ofer Pasternak

**Affiliations:** 1 Department of Psychiatry, Mass General Brigham, Harvard Medical School, Boston, Massachusetts; 2 IBM Research, T.J. Watson Research Laboratory, Yorktown Heights, New York; 3 Department of Psychiatry and Behavioral Sciences, University of California, San Francisco; 4 San Francisco Veterans Affairs Health Care System, San Francisco, California; 5 Department of Psychiatry, Icahn School of Medicine at Mount Sinai, New York, New York; 6 Mental Illness Research, Education and Clinical Center, James J. Peters VA Medical Center, New York, New York; 7 Department of Environmental Medicine and Public Health, Icahn School of Medicine at Mount Sinai, New York, New York; 8 Mindich Child Health and Development Institute, Icahn School of Medicine at Mount Sinai, New York, New York; 9 Division of Pharmacoepidemiology and Pharmacoeconomics, Department of Medicine, Brigham and Women’s Hospital, Boston, Massachusetts; 10 Department of Psychiatry, Hotchkiss Brain Institute, University of Calgary, Calgary, Alberta, Canada; 11 Department of Radiology, Brigham and Women’s Hospital, Harvard Medical School, Boston, Massachusetts; 12 Department of Software Engineering and Information Technology, École de Technologie Supérieure, Université du Québec, Montréal, Quebec, Canada; 13 Orygen, Parkville, Victoria, Australia; 14 Centre for Youth Mental Health, The University of Melbourne, Parkville, Victoria, Australia; 15 Department of Psychiatry, Yale School of Medicine, New Haven, Connecticut; 16 Connecticut Mental Health Center, New Haven; 17 Departments of Psychiatry and Biobehavioral Sciences and Psychology, Semel Institute for Neuroscience and Human Behavior, University of California, Los Angeles; 18 Department of Psychiatry, Donald and Barbara Zucker School of Medicine at Hofstra/Northwell, Hempstead, New York; 19 Institute of Behavioral Science, Feinstein Institutes for Medical Research, Manhasset, New York

## Abstract

**Importance:**

While remission from clinical high risk (CHR) for psychosis is a favorable outcome, it is not well characterized over time.

**Objective:**

To examine remission incidence, prevalence, and stability, and their association with demographic, clinical, medication, and cognitive variables, comparing 2 commonly used definitions.

**Design, Setting, and Participants:**

This cohort study examined data from individuals aged 12 to 30 years at CHR in the North American Prodromal Longitudinal Study 3, collected from 9 sites across the US from February 2015 to November 2018. Statistical analyses were conducted between January 2023 and May 2025.

**Exposure:**

CHR status using 2 definitions: (1) a symptoms-only definition based on the positive symptoms from the Scale of Prodromal Symptoms and (2) a symptoms-and-function definition based on positive symptoms and the modified Global Assessment of Functioning.

**Main Outcomes and Measures:**

The primary outcomes were remission incidence, prevalence, and stability for 7 follow-up visits over 2 years. Associations of remission with age, sex at birth, race, antipsychotic and antidepressant medication, history of trauma, and cognitive performance were determined using mixed-effects logistic regression.

**Results:**

The sample included 692 individuals (mean [SD] age, 18.7 [4.1] years; 319 female [46%]) at baseline, with 614 completing at least 1 follow-up. For the symptoms-only definition, 7% (95% CI, 5%-10%) met remission criteria after 2 months, 34% (95% CI, 31%-38%) met remission criteria at least once during the study, and 26% (95% CI, 22%-29%) met criteria at their last visit. The symptoms-and-function definition was associated with a lower remission incidence and prevalence, with 4% (95%CI, 2%-5%) meeting remission criteria after 2 months, 21% (95% CI, 18%-24%) meeting criteria at least once, and 15% (95% CI, 13%-18%) meeting criteria at their last visit. Under the symptoms-only definition, 83 of 153 individuals at CHR with at least 1 follow-up after remission (54%; 95% CI, 46%-62%) were stable remitters. Under the symptoms-and-function definition, 43 of 91 individuals (47%; 95% CI, 37%-58%) were stable remitters. The chance of staying in remission rose drastically once a person had more than 1 previous recorded remission visit. Higher functioning was associated with higher likelihood of remission (current score for symptoms only: OR, 1.04; 95% CI, 1.01-1.08; current score for symptoms and function: OR, 1.08; 95% CI, 1.02-1.14). More symptoms at baseline was associated with a lower likelihood of remission (general symptoms for symptoms only: OR, 0.77; 95% CI, 0.70-0.84; general symptoms for symptoms and function: OR, 0.80; 95% CI, 0.69-0.92).

**Conclusions and Relevance:**

These findings suggest that CHR status is a dynamic state and that vulnerability can persist even after functional remission. Hence, continued follow-up and facilitated reengagement with clinical services after remission are essential.

## Key Points

**Question:**

What are the incidence, prevalence, and stability of remission in individuals at clinical high risk (CHR) for psychosis, and how are these associated with clinical and other characteristics?

**Findings:**

In this cohort study of 692 individuals at CHR, 34% achieved symptoms-only remission and 21% achieved symptom-and-function remission within 2 years. Overall, 54% of remitters were stable under the symptoms-only definition, 47% of remitters were stable under the symptoms-and-function definition, and the chance of staying in remission rose drastically once a person had more than 1 previous recorded remission visit; remission was associated with higher functioning and fewer symptoms at baseline.

**Meaning:**

These findings suggest that CHR status is a fluctuating condition, regardless of how it is defined, and more than 2 assessments are recommended for sustained remission.

## Teaser

This cohort study compares incidence, prevalence, and stability of remission, and associations with clinical and demographic factors among individuals at clinical high risk (CHR) for psychosis using 2 common definitions of CHR.

## Introduction

Clinical high risk (CHR) for psychosis, at-risk mental state, and ultra–high risk are overlapping frameworks capturing the period before psychosis onset.^1,2^ They are characterized by the presentation of 1 or more of the following: (1) attenuated positive psychotic symptoms; (2) brief, limited intermittent psychotic symptoms; or (3) genetic risk accompanied by functional decline. Most CHR research has concentrated on characterizing and predicting transition to psychosis to inform treatment development and forestall or postpone this transition. However, most individuals at CHR do not transition.^3,4^ While some will experience persistent attenuated symptoms and other negative outcomes, including psychiatric comorbidities and psychosocial impairments,^5^ others will show improvements in their symptoms and functioning over time.

Consequently, researchers have expanded their focus to include remission from CHR-related symptoms.^6,7^ Early identification of individuals likely to remit could optimize treatment allocation, including clinical trial participation, and understanding remission-related factors may inform preventive strategies and interventions.^8^

Current literature reports a wide range of remission rates,^5,9^ which could be attributed to several factors. First, the definition of remission across studies is inconsistent. While most definitions focus on positive symptom remission (symptoms only), others include additional functioning criteria (symptoms and function).^10^ Studies using functioning criteria report fewer individuals meeting remission,^11^ although a systematic comparison of the 2 definitions has not been conducted. Second, studies have varied substantially in follow-up duration and visit frequency; this is critical because evidence suggests that relapses can occur after remission^12,13^ or remission can be sustained.^14,15^ Thus, multiple follow-ups are needed to better capture remission patterns. Lastly, cohort-specific demographic, clinical, medication, and cognitive variables might be associated with remission, although these have not been studied systematically.

The present study investigated remission in the context of these 3 factors by leveraging longitudinal data from the North American Prodromal Longitudinal Study (NAPLS-3). The large sample and standardized, frequent assessments enabled dynamic evaluations of remission status, its persistence, and associations with measures of interest. Using both symptoms-only and symptoms-and-function definitions, we assessed (1) the incidence of remission (ie, the percentage of individuals who were newly remitted at a visit) and prevalence of remission (ie, the total percentage of individuals who were in remission at a visit across 2 years), (2) the stability of remission over 2 years, and (3) associations with demographic, clinical, medication, and cognitive variables.

## Methods

### Sample

This cohort study follows the Strengthening the Reporting of Observational Studies in Epidemiology (STROBE) reporting guideline. Data from 698 individuals at CHR aged 12 to 30 years collected at 9 US sites from February 2015 to November 2018 in the NAPLS-3 study were obtained from the National Institute of Mental Health Data Archive. The study was approved by local institutional review boards and written informed consent was obtained. CHR status was determined using the Structured Interview for Psychosis-Risk Syndromes (SIPS).^16^ We excluded 6 participants who met CHR status based on genetic risk with functional decline only, because their positive symptoms already met remission criteria at baseline. All available data from baseline and follow-up visits at months 2, 4, 6, 8, 12, 18, and 24 were included.

### Defining Remission

We compared 2 remission definitions. The first, symptoms-only remission,^17^ classified individuals at CHR as remitted if all 5 positive symptom ratings from the Scale of Prodromal Symptoms, part of the SIPS, were less than 3. The second definition, symptoms and function,^12,13,18,19^ required that all 5 Scale of Prodromal Symptoms positive symptom ratings were less than 3 and a Global Assessment of Functioning (GAF) total score was greater than 60. This GAF cutoff was chosen based on previous studies.^20,21,22^

### Statistical Analysis

Analyses were conducted using Excel version 16.0300 (Microsoft), SPSS version 29.0.0 (IBM), Prism10 (Dotmatics), and R version 4.0.3 (R Project for Statistical Computing) between January 2023 and May 2025. Missing data were not imputed (see eFigure 1 and eTable 1 in Supplement 1 for per-visit sample sizes). Individuals who transitioned to psychosis were labeled as converters for all subsequent visits to avoid overestimating remission prevalence.^23^

We conducted all statistical analyses twice, using the (1) symptoms-only and (2) symptoms-and-function remission definitions. Throughout the results, we report absolute counts, the percentage of individuals remitted relative to the individuals with data available at that visit, and the 95% CIs for these percentages (Wald method; minimum point 0). Group differences were considered significant if their 95% CI did not overlap. For mixed-effects logistic regression models, logistic regression models, and analysis of variance, confidence intervals and *P* values were considered. A 2-sided *P* < .05 after correction for multiple comparisons using false discovery rate was considered significant.

#### Incidence, Probability, and Prevalence of Remission

Incidence was calculated as the percentage of individuals newly remitted at each visit out of those eligible for first-time remission. Probability of first remission was calculated as the percentage of newly remitted individuals out of those with available data. To account for varying follow-up intervals, both incidence and probability were standardized to a 2-month period.

Among individuals at CHR with at least 1 follow-up, we calculated overall the percentage who (1) achieved remission at any point during the 2-year follow-up (regardless of final visit) and (2) were in remission at their last recorded visit. The prevalence of remission for each visit was calculated as the percentage of individuals in remission among all participants with available data.

#### Stability of Remission

A stable remitter was defined as someone who met remission criteria at all remaining visits after initial remission. An unstable remitter no longer met remission criteria or transitioned to psychosis after remission.

Among individuals at CHR with at least 1 follow-up after remission, we calculated the percentage of overall stable remitters. We also calculated the percentages of first-time remitters, stable remitters, and unstable remitters for all follow-up visits.

We calculated overall remission probability based on the number of prior remission visits (none, 1, 2, or more than 2 visits). Then, for each visit, we grouped individuals by their number of previous remission visits and calculated remission prevalence separately for each subgroup.

#### Association With Demographic, Clinical, Medication, and Cognitive Variables

We stratified the sample by age (younger or older than median age at baseline), sex at birth (female and male), race (Asian, Black or African American, individuals of more than 1 race, White, and excluded due to small samples [American Indian or Alaska Native and Native Hawaiian or Pacific Islander]), antipsychotic use (ever or never used before or at that visit), antidepressant use (ever or never used before or at that visit), and history of trauma (with or without, based on emotional neglect, psychological, physical, or sexual abuse). We calculated the remission prevalence for each subgroup at each follow-up visit. Race was self-reported using categories defined by the investigators and was included to allow the characterization of remission trajectories based on potentially relevant variables.

We examined how baseline age, sex at birth, race, antipsychotic and antidepressant use, trauma history (Childhood Trauma and Abuse Scale^24^), cognitive functioning, GAF, and SIPS scores were associated with remission. Cognitive functioning was assessed using 3 MATRICS (Measurement and Treatment Research to Improve Cognition in Schizophrenia) battery scores (Hopkins Verbal Learning Test-Revised, Brief Assessment of Cognition in Schizophrenia, and Letter-Number Span)^25^ and 2 Wechsler Abbreviated Scale of Intelligence scores (matrix and vocabulary).^26^ Mixed-effects logistic regression models were used to assess the association of each baseline variable and remission across visits, with months since baseline as a fixed effect and participants as a random effect. Results are reported as odds ratios (ORs) with 95% CIs and significance levels. Logistic regression models were used to compare baseline variables between stable vs unstable remitters, without additional fixed or random effects.

#### Sensitivity Analyses

First, we explored remission incidence and prevalence by visit using varying GAF cutoffs (30-80). Second, we examined remission rates by visit among individuals without baseline antipsychotic use. Third, we assessed associations of the number of follow-up visits (0-7, including conversion visits) and baseline variables (age, sex at birth, race, GAF, SIPS, antipsychotic and antidepressant use, trauma history, and cognitive functioning) using analysis of variance. Fourth, we estimated remission prevalence by visit across the 9 study sites to identify outliers and assess whether site was associated with remission.

## Results

Table 1 and eTable 2 in Supplement 1 summarize demographic, clinical, medication, and cognitive data by visit. We analyzed 692 individuals at baseline (mean [SD] age, 18.7 [4.1] years; 319 female [46%]). Across the 7 follow-up visits, sample sizes ranged from 245 to 484 participants, with 614 participants completing at least 1 follow-up.

**Table 1.** Demographic and Clinical Information at Each Visit^a^

Characteristic	Participants by time period, No. (%)							
	Baseline (n = 692)	Month 2 (n = 484)	Month 4 (n = 434)	Month 6 (n = 394)	Month 8 (n = 396)	Month 12 (n = 365)	Month 18 (n = 286)	Month 24 (n = 245)
Age, mean (SD), y	18.7 (4.1)	18.8 (3.9)	19.2 (4.1)	19.1 (3.9)	19.4 (4.1)	19.9 (4.2)	20.5 (4.2)	21.0 (4.1)
Sex								
Female	319 (46)	231 (48)	202 (47)	181 (46)	184 (46)	168 (46)	135 (47)	115 (47)
Male	373 (54)	253 (52)	232 (53)	213 (54)	212 (54)	197(54)	151 (53)	130 (53)
Race								
American Indian or Alaska Native	14 (2)	12 (2)	10 (2)	11 (3)	11 (3)	9 (2)	9 (3)	6 (2)
Asian	81 (12)	53 (11)	46 (11)	42 (11)	46 (12)	38 (10)	30 (10)	26 (11)
Black or African American	80 (12)	59 (12)	49 (11)	45 (11)	43 (11)	42 (12)	37 (13)	32 (13)
Missing	1 (<1)	1 (<1)	1 (<1)	0	0	1 (<1)	1 (<1)	0
Native Hawaiian or Pacific Islander	2 (<1)	2 (<1)	1 (<1)	2 (1)	1 (<1)	2 (1)	1 (<1)	1 (<1)
>1 Race	90 (13)	63 (13)	55 (13)	54 (14)	58 (15)	47 (13)	32 (11)	35 (14)
White	424 (61)	294 (61)	272 (63)	240 (61)	237 (60)	226 (62)	176 (62)	145 (59)
Antipsychotic use	216 (31)	106 (22)	94 (22)	88 (22)	89 (22)	78 (21)	59 (21)	47 (19)
Antidepressant use	331 (48)	155 (32)	157 (36)	146 (37)	145 (37)	127 (35)	98 (34)	84 (34)
Trauma								
Trauma occurrence	342 (49)	248 (51)	212 (49)	196 (50)	197 (50)	176 (48)	136 (48)	111 (45)
No. of traumas, mean (SD)	1.0 (1.2)	0.9 (1.1)	1.0 (1.2)	0.9 (1.1)	0.9 (1.1)	1.0 (1.2)	1.0 (1.2)	1.0 (1.2)
Trauma impact, mean (SD)	3.6 (4.7)	3.4 (4.5)	3.7 (4.8)	3.5 (4.5)	3.5 (4.6)	3.8 (4.8)	3.9 (4.9)	3.8 (4.7)
MATRICS battery, mean (SD)								
Hopkins Verbal Learning Test-Revised	26.4 (5.2)	26.3 (5.1)	26.8 (5.1)	28.5 (4.7)	27.5 (5.5)	NA	NA	NA
BACS	54.3 (13.3)	60.7 (14.6)	62 (15.3)	64 (14.9)	65.8 (16.3)	NA	NA	NA
Letter-number span	14.6 (3.7)	NA	NA	NA	15.1 (3.6)	NA	NA	NA
Wechsler Abbreviated Scale of Intelligence, mean (SD)								
Matrix	20.9 (4.4)	NA	NA	NA	NA	NA	NA	NA
Vocabulary	38.2 (6.5)	NA	NA	NA	NA	NA	NA	NA
GAF, mean (SD)								
Current score	51.0 (12.0)	53.7 (12.4)	54.6 (13.5)	55.7 (13.5)	57.3 (13.6)	58.8 (12.6)	60 (12.5)	59.9 (14.2)
Highest score in the last year	57.9 (13.8)	57.6 (14.0)	57.2 (13.5)	57.0 (13.0)	56.1 (13.7)	53.9 (12.5)	57.1 (13.1)	58.2 (13.2)
Past-month SIPS, mean (SD)								
Positive	12.9 (3.4)	10.8 (3.9)	10.0 (4.4)	9.2 (4.4)	8.5 (4.5)	7.9 (4.3)	7.4 (4.5)	6.9 (4.4)
Negative	12.1 (6.3)	11.0 (6.4)	10.3 (6.4)	9.7 (6.2)	9.5 (6.6)	8.9 (6.6)	8.5 (6.2)	8.1 (6.2)
Disorganized	5.2 (3.2)	4.6 (3.0)	4.6 (3.2)	4.2 (3.2)	4.0 (3.2)	3.8 (3.3)	3.6 (3.2)	3.6 (3.0)
General	9.4 (4.2)	8.4 (4.4)	7.6 (4.4)	7.4 (4.5)	6.8 (4.6)	6.7 (4.4)	6.5 (4.5)	6.8 (4.5)

Abbreviations: BACS, Brief Assessment of Cognition in Schizophrenia; GAF, Global Assessment of Functioning; MATRICS, Measurement and Treatment Research to Improve Cognition in Schizophrenia; NA, not applicable; SIPS, Structured Interview for Psychosis-Risk Syndromes.

^a^ The numbers in Table 1 are based on the data available for each visit and that the North American Prodromal Longitudinal 3 Study did not collect regular follow-up visit information for individuals after they converted. However, 2 participants in month 2 and 1 participant in month 6 converted shortly after their regular study visit. Information for their regular visits is included in this table, but these participants are labeled as converted at these visits in all other tables and figures. Information regarding sex, race, and trauma is only based on information provided at the baseline visit. Race was self-reported using categories defined by the investigators. For further analyses, we excluded individuals identifying as American Indian or Alaska Native, missing race, and Native Hawaiian or Pacific Islander due to small sample sizes. Age, antipsychotic use, antidepressant use, GAF, and SIPS were collected at each follow-up visit. Cognition was collected at some follow-up visits and is presented in the table accordingly. For more detailed medication information, see eTable 2 in Supplement 1.

### Incidence, Probability, and Prevalence of Remission

#### Incidence and Probability of Remission by Visit

**Figure 1.**
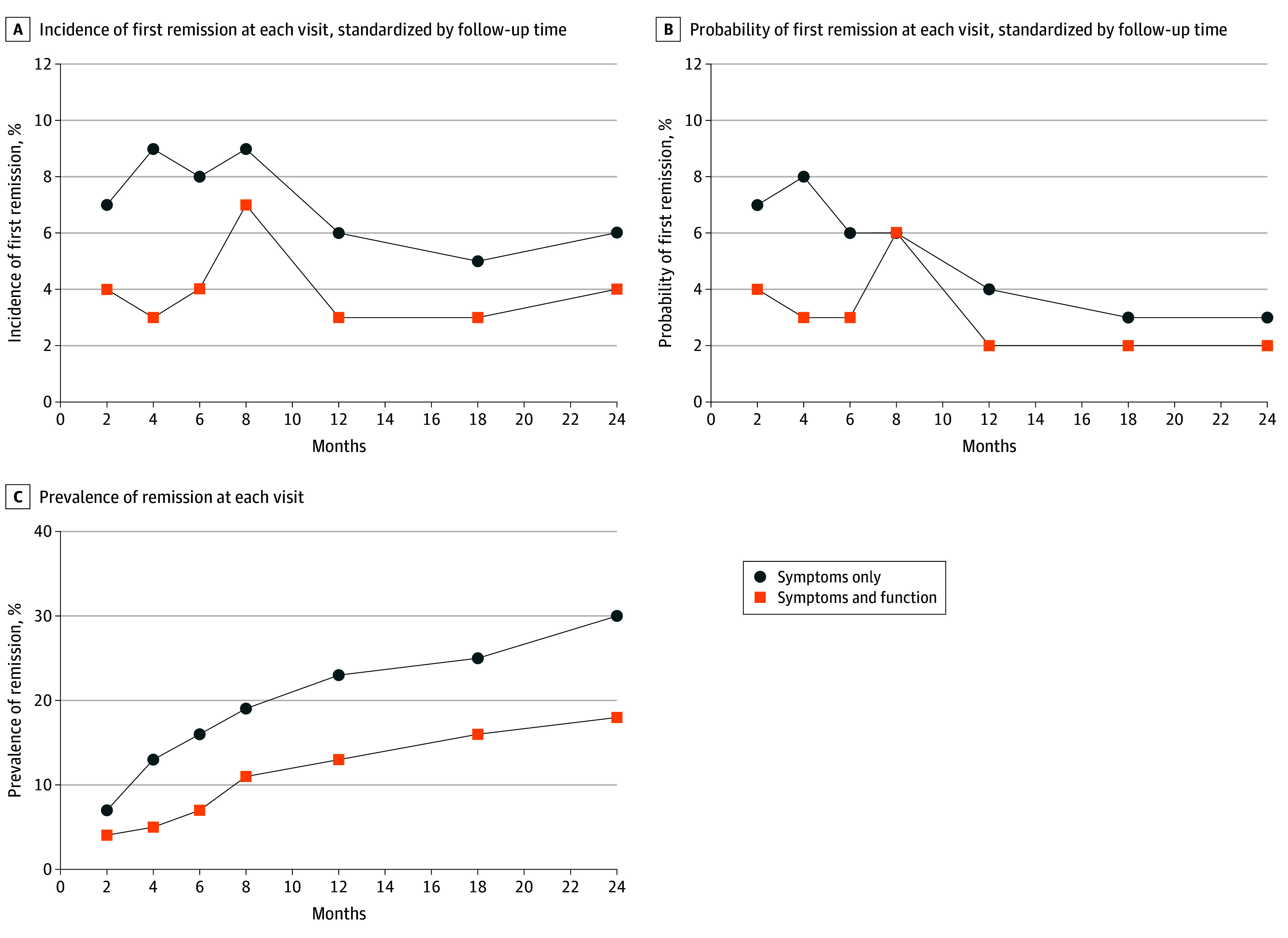
Incidence and Prevalence of Remission at Each Follow-Up Visit A, Incidence of first remission based on who was eligible to achieve first remission at this visit. B, The probability of first remission based on everyone with available data for this visit (including converters). C, The prevalence of remission based on the available data for this visit. Individuals who converted at or before a visit were included in the analyses as nonremitters. Panels A and B report measures standardized by a 2-month follow-up time. For unstandardized measures, see eFigure 2 in Supplement 1. To see absolute counts, percentage of individuals remitted relative to the individuals with data available, and 95% CIs for these percentages, see eTable 3, eTable 4, and eTable 6 in Supplement 1.

The standardized remission incidence was consistent across follow-ups (5%-9% for symptoms-only and 3%-7% for symptoms and function) (Figure 1A and eTable 3 in in Supplement 1). The incidences were higher for the symptoms-only definition compared with the symptoms-and-function definition, and significantly so at month 4. For unstandardized measures and probability of first remission at each visit, see eFigure 2 and eTable 4 in Supplement 1. For status information available at each visit, see eFigure 1, eTable 1 and eTable 5 in Supplement 1).

#### Overall Prevalence of Remission

Of the 614 participants at CHR with 1 follow-up, using the symptoms-only definition, 211 (34%; 95% CI, 31%-38%) were in remission at least once, compared with 130 participants (21%; 95% CI, 18%-24%) using the symptoms-and-function definition (eFigure 3 in Supplement 1). At their last recorded visit, 159 individuals at CHR (26%; 95% CI, 22%-29%) were in remission using the symptoms-only definition compared with 94 participants (15%; 95% CI, 13%-18%) using the symptoms-and-function definition (eFigure 3 in Supplement 1).

#### Prevalence of Remission by Visit

Remission prevalence was significantly higher for the symptoms-only definition compared with the symptoms-and-function definition at all follow-up visits except month 2 (Figure 1C and eTable 6 in Supplement 1). Of the 614 participants at CHR with 1 follow-up, using the symptoms only definition, 36 participants (7%; 95% CI, 5%-10%) met remission criteria after 2 months, compared with 18 participants (4%; 95% CI, 2%-5%) using the symptoms-and-function definition. By month 24, prevalence reached 30% (95% CI, 25%-35%) for symptoms only and 18% (95% CI, 14%-23%) for symptoms and function.

### Stability of Remission

#### Overall Stability of Remission

Under the symptoms-only definition, 83 of 153 individuals at CHR with at least 1 follow-up after remission (54%; 95% CI, 46%-62%) were stable remitters. Among 70 unstable remitters, 65 (93%) met CHR criteria again and 5 (7%) converted to psychosis. Under the symptoms-and-function definition, 43 of 91 individuals (47%; 95% CI, 37%-58%) were stable remitters. Of the 48 unstable remitters, 46 (96%) met CHR criteria again and 2 (4%) converted (eFigure 3 in Supplement 1).

#### Stability of Remission by Visit

**Figure 2.**
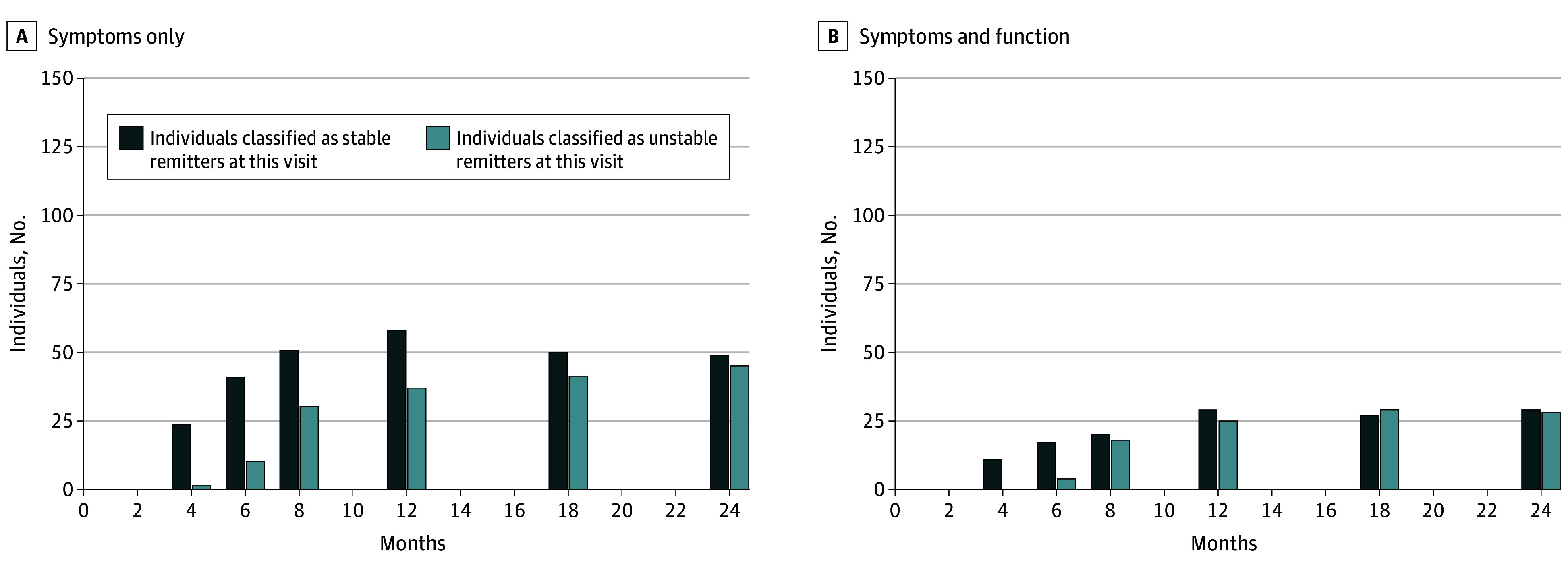
Stability of Remission at Each Visit A stable remitter was defined as someone who did not meet clinical high risk (CHR) criteria again or transitioned to psychosis after a previous remission. An unstable remitter was defined as someone who met CHR criteria again or transitioned to psychosis after a previous remission. For absolute counts, the percentage of individuals who were first time, stable, and unstable remitters relative to all who had remitted up to that follow-up visit, and 95% CIs for these percentages, see eTable 7 in Supplement 1.

For the symptoms-only definition, unstable remitters increased from 2% (95% CI, 0%-5%) at month 4 to 12% (95% CI, 7%-18%) for unstable remitters who were currently remitted and 25% (95% CI, 17%-32%) for unstable remitters who were not currently remitted at month 24. For the symptoms-and-function definition, the increase was from 0% to 10% (95% CI, 4%-17%) for unstable remitters who were currently remitted and 26% (95% CI, 16%-36%) for unstable remitters who were not currently remitted over the same period. The percentage of unstable remitters did not differ significantly between definitions (Figure 2 and eTable 7 in Supplement 1).

#### Association of Previous Remission Visits With the Probability and Prevalence of Remission

**Figure 3.**
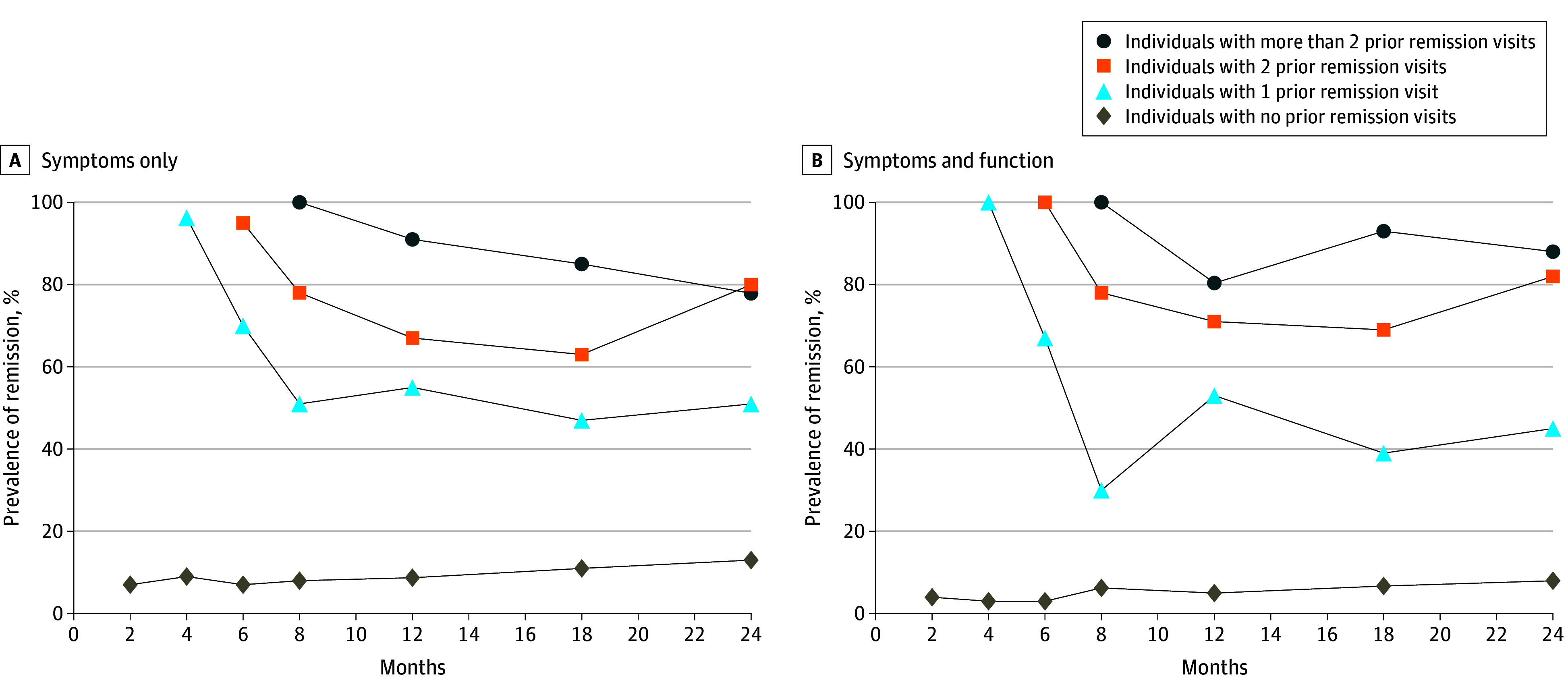
Prevalence of Remission at Each Visit, Depending on Previous Remission Visits For absolute counts, the percentage of individuals remitted relative to the individuals with data available, and 95% CIs for these percentages, see eTable 8 and eTable 9 in Supplement 1.

Detailed trends by visit are shown in Figure 3. For each follow-up visit, we split individuals into groups with no previous remission visit, 1 previous remission visit, 2 previous remission visits, or more than 2 previous remission visits. We calculated the prevalence of remission for the groups separately. Individuals who converted at or before a visit were included in the analysis as nonremitters. For the symptoms-only definition, across visits, the probability of remission given 0 previous remission visits was 9% (95% CI, 8%-10%), 1 previous remission visit was 59% (95% CI, 52%-66%), 2 previous remission visits was 72% (95% CI, 64%-80%), and 3 or more previous remission visits was 87% (95% CI, 81%-93%). The probabilities for the symptoms-and-function definition across visits were 5% (95% CI, 4%-6%) for 0 previous remission visits, 47% (95% CI, 39%-56%) for 1 previous remission visit, 81% (95% CI, 70%-92%) for 2 previous remission visits, and 92% (95% CI, 85%-100%) for 3 or more previous remission visits. We can see that the prevalence of remission increased significantly for both definitions with 1 or more previous remission visits recorded. For the symptoms-only definition, the remission prevalence for specific visits varied between 47% (95% CI, 30%-64%) and 96% (95% CI, 88%-100%) for individuals with 1 previous remission visit, 63% (95% CI, 43%-82%) and 95% (86%-100%) for individuals with 2 previous remission visits, and 78% (95% CI, 65%-92%) and 100% for individuals with more than 2 previous remission visits. For the symptoms-and-function definition, the prevalence for specific visits varied between 30% (95% CI, 10%-50%) and 100% for individuals with 1 previous remission visit, 69% (95% CI, 46%-92%) and 100% for individuals with 2 previous remission visits, and 80% (95% CI, 60%-100%) to 100% for individuals with more than 2 previous remission visits. For absolute counts, the percentage of individuals remitted relative to the individuals with data available, and 95% CIs for these percentages, see eTable 8 and eTable 9 in Supplement 1.

### Association With Demographic, Clinical, Medication, and Cognitive Variables

#### Prevalence of Remission and Demographic, Clinical, Medication, and Cognitive Variables by Visit

Baseline age (eFigure 4 in Supplement 1), sex at birth (eFigure 5 in Supplement), race (eFigure 6 in Supplement 1), antipsychotic use (eFigure 7 in Supplement 1), and history of trauma (eFigure 8 in Supplement 1) were not associated with remission prevalence. Not taking antidepressants prior to or at month 8 was associated with a higher remission prevalence for both definitions, but not at any other visit (eFigure 9 in Supplement 1).

#### Overall Prevalence and Stability of Remission and Baseline Variables

Higher GAF scores were associated with a higher likelihood of remission across visits (current score for symptoms only: OR, 1.04; 95% CI 1.01-1.08; current score for symptoms and function: OR, 1.08; 95% CI, 1.02-1.14), while higher positive (symptoms only: OR, 0.67; 95% CI, 0.59-0.76; symptoms and function: OR, 0.83; 95% CI, 0.69-1.00), negative (symptoms only: OR, 0.92; 95% CI, 0.86-0.98; symptoms and function: OR, 0.86; 95% CI, 0.78-0.96), disorganized (symptoms only: OR, 0.71; 95% CI, 0.62-0.81; symptoms and function: OR, 0.72; 95% CI, 0.58-0.90), and general (symptoms only: OR, 0.77; 95% CI, 0.70-0.84; symptoms and function: OR, 0.80; 95% CI, 0.69-0.92) SIPS scores were significantly associated with a lower likelihood of remission across visits (Table 2). We did not see any significant differences between stable and unstable remitters after correction for multiple comparisons (eTable 10 in Supplement 1).

**Table 2.** Association of Baseline Variables With Remission^a^

Variable	Symptoms only		Symptoms and function	
	Remission, OR (95% CI)	*P* value	Remission, OR (95% CI)	*P* value
Age	1.04 (0.94-1.15)	.48	0.99 (0.86-1.13)	.85
Female sex	1.90 (0.85-4.28)	.12	1.35 (0.43-4.21)	.60
Race^b^				
Asian	3.12 (0.82-11.83)	.09	0.99 (0.03-39.10)	>.99
Black or African American	0.51 (0.12-2.20)	.37	0.27 (0.00-29.91)	.59
More than 1 race	0.82 (0.21-3.16)	.77	0.67 (0.02-23.43)	.82
Antipsychotic use	0.99 (0.40-2.41)	.98	0.90 (0.25-3.17)	.26
Antidepressant use	0.54 (0.24-1.21)	.14	0.52 (0.17-1.63)	.87
Trauma				
Trauma occurrence	0.68 (0.30-1.51)	.34	0.66 (0.21-2.05)	.47
Number of traumas	0.84 (0.60-1.19)	.34	0.87 (0.53-1.43)	.59
Trauma impact	0.93 (0.86-1.02)	.13	0.96 (0.85-1.08)	.51
MATRICS				
Hopkins Verbal Learning Test-Revised	1.02 (0.94-1.10)	.72	1.01 (0.90-1.13)	.84
Brief Assessment of Cognition in Schizophrenia	1.02 (0.99-1.05)	.29	1.03 (0.98-1.08)	.25
Letter-number span	1.10 (0.97-1.23)	.13	1.08 (0.90-1.28)	.41
Wechsler Abbreviated Scale of Intelligence				
Matrix score	0.94 (0.85-1.03)	.18	0.99 (0.87-1.12)	.84
Vocabulary score	0.99 (0.93-1.06)	.88	1.00 (0.92-1.10)	.92
GAF				
Current score	1.04 (1.01-1.08)	.01^c^	1.08 (1.02-1.14)	.004^c^
Highest score in the last year	1.04 (1.01-1.07)	.01^c^	1.08 (1.03-1.13)	.002^c^
SIPS				
Positive	0.67 (0.59-0.76)	<.001^c^	0.83 (0.69-1.00)	.048^d^
Negative	0.92 (0.86-0.98)	.01^c^	0.86 (0.78-0.96)	.004^c^
Disorganized	0.71 (0.62-0.81)	<.001^c^	0.72 (0.58-0.90)	.004^c^
General	0.77 (0.70-0.84)	<.001^c^	0.80 (0.69-0.92)	.001^c^

Abbreviations: GAF, Global Assessment of Functioning; MATRICS, Measurement and Treatment Research to Improve Cognition in Schizophrenia; OR, odds ratio; SIPS, Structured Interview for Psychosis-Risk Syndromes.

^a^ Table 2 is based on baseline data for all individuals with at least 1 follow-up visit (614 participants). Please note that not all individuals had information for all variables.

^b^ ORs for race were calculated with White individuals as the reference group.

^c^ Represents statistically significant group differences after correction for multiple comparisons using false discovery rate.

^d^ Represents statistically significant group differences.

### Sensitivity Analyses

Higher GAF cutoffs were associated with lower remission incidence and prevalence, although patterns remained consistent (eFigure 10 in Supplement 1). Incidences and prevalences of remission among individuals without baseline antipsychotic use were similar to the full sample, supporting generalizability of our findings (eFigure 11 in Supplement 1). Participants with fewer follow-ups had lower baseline GAF, higher positive SIPS scores, and more antipsychotic use, but no other significant baseline differences (eTable 11 in Supplement 1). Remission prevalence did not significantly vary by site (eTable 12 in Supplement 1).

## Discussion

Using the large, longitudinal NAPLS-3 dataset, this cohort study examined the incidence, prevalence, and stability of remission in individuals at CHR over 2 years. While remission incidence was consistent, the prevalence increased across the 24 months’ follow-up and was significantly lower when using the symptoms-and-function compared with the symptoms-only definition. Only 54% of remitters were stable, indicating that the CHR status is a fluctuating condition. Fluctuations were similar across both remission definitions. However, the chance of staying in remission rose drastically once a person had 1 or more previous recorded remission visits. Higher likelihood of remission was associated with higher functioning and fewer symptoms at baseline, but no other baseline variables. Together these findings point toward potential benefits of follow-ups and engagement in clinical services for participants in remission for at least 6 months after initial remission.

### Incidence and Prevalence for the Symptoms-Only Definition

Remission incidence (5%-9% across all follow-ups) and prevalence (34%) fell within the range of previous studies,^5^ underscoring the persistent nature of CHR for most individuals.^9,14^ Our study was limited to 24 months’ follow-up; however, other studies report that initial remission can happen up to 10 years after CHR identification.^27^

### Stability of Remission

Only 54% of remitters were stable, demonstrating how dynamic remission is. Nevertheless, prevalence increased after previous remission visits, suggesting that definitions of stable remission should depend on how long participants remain in remission, rather than on being in remission at the last study visit. Our findings tentatively support a stable remission definition based on at least 6 months of sustained remission, given that the likelihood of staying in remission increased to 72% (symptoms only) and 81% (symptoms and function) after 2 previous remission visits and 87% (symptoms-only) and 92% (symptoms-and-function) after 3 or more previous remission visits. This finding aligns with NAPLS-1, which considered a 6-month period as sustained.^15^ However, the high percentage of unstable remitters suggests the need for multiple follow-up visits to determine stable remission and facilitated reentry into clinical services.

### Inclusion of Function in the Remission Definition

It is not surprising that overall prevalence was lower with the symptoms-and-function definition, which is aligned with previous smaller studies.^11,28,29^ However, neither the remission stability, nor the association of remission with time was different between the 2 definitions. Sensitivity analyses corroborate this finding, showing comparable remission patterns for different GAF cutoffs. However, because the GAF ratings are associated with symptom severity,^30^ future studies should consider more function-specific tools to track remission. While only a few individuals converted to psychosis postremission, this number was not significantly smaller for the symptoms-and-function definition. Together these findings suggest that the addition of GAF may not be necessary for clinical studies focused on predicting or improving psychosis symptoms, and that even a single symptomatic remission visit is a sufficient marker of reduced risk of later conversion.^28^

### Association With Demographic, Clinical, Medication, and Cognitive Variables

Higher remission likelihood was significantly associated with higher functioning and fewer baseline symptoms only. These results were expected and are similar to a prior study highlighting the predictive role of baseline functioning for 12-month remission.^7^

While not statistically significant, the descriptive analyses showed lower remission prevalence for females, individuals with a history of trauma, and for Black and African American individuals. The association of sex at CHR outcomes is still debated, with our findings being in line with a previous meta-analysis that found no association of sex with symptom changes in CHR.^9^ Future research should strive for ethnic and racial balance to examine potential associations with symptoms and functioning in individuals at CHR^31^ and the interplay of racial and ethnic minority status with perceived discrimination.^32^

Consistent with other studies,^11,33^ we also found no association of antipsychotic use with remission, and not using antidepressants was only associated with an increased likelihood of remission at month 8. These results should be interpreted cautiously because medication is often prescribed for more severe symptoms,^34^ which are associated with a lower likelihood of remission. Also, nonadherence is common and a major confounder in outcome studies.^35^ Lastly, our medication-related analyses did not account for dosage, duration, or other pharmacological and nonpharmacological treatments.

While not significant when correcting for multiple comparison, symptoms-only stable remitters showed better Brief Assessment of Cognition in Schizophrenia performance compared with unstable remitters. These results align with earlier work, showing that lower cognitive functioning, specifically in verbal learning and executive functioning as captured by the Brief Assessment of Cognition in Schizophrenia, has been associated with poor outcomes for individuals at CHR.^31^

### Limitations

This study has limitations. The main limitation was missing data for many participants at 1 or more follow-ups. As in past studies,^36^ we found few baseline demographic differences associated with missing visits (see sensitivity analyses), although lower baseline functioning was associated with fewer follow-ups. This finding suggests assumptions about random missingness and dropouts warrant further testing. Missing data also reduced sample sizes for stability and prior remission analyses (Figure 3). Furthermore, findings require external validation beyond the NAPLS-3 dataset because it may not be representative of other cohorts that differ by geographic location, criteria used to define CHR, or time of ascertainment. Future research should also consider more dimensional approaches,^33^ finer-grained instruments to assess functioning,^34,35^ as well as more detailed studies of functional remission. Additionally, several variables not included in our analyses should be considered, such as comorbidities and psychosocial factors and treatments.^37^

## Conclusions

In this cohort study of individuals at CHR from NAPLS-3, we found that symptomatic and functional remission was less common than symptomatic remission alone. While the incidence of remission remained stable, its prevalence increased over the 24-month follow-up period. Regardless of the definition used, only one-half of remitters were stable, indicating that remission is a dynamic state and that vulnerability can persist even after functional remission. However, the likelihood of remaining in remission rose substantially once a person had more than 2 previously recorded remission visits, suggesting that at least 6 months of follow-up are recommended to confirm sustained remission. Higher remission likelihood was associated with higher functioning and lower baseline symptoms, but not with other sociodemographic, medication, cognitive, or trauma variables. Future research should explore broader risk factors, as well as potential complex interactions among them. Gaining deeper insight into the incidence, prevalence, and stability of remission is an essential step toward developing individualized prediction models and stratification approaches for clinical monitoring and treatment of individuals at CHR.

## Notes

Supplement 1. **eFigure 1.** Status Information Available at Each Visit **eTable 1.** Status Information Available at Each Visit **eTable 2.** Medication Information at Each Visit **eTable 3.** Incidence of Remission at Each Visit, Standardized by Follow-Up Time **eFigure 2.** Incidence and Prevalence of Remission for Each Follow-Up Visit (Non-Standardized) **eTable 4.** Probability of First Remission at Each Visit, Standardized by Follow-Up Time **eTable 5.** Status Information at the Last Recorded Visit **eFigure 3.** Overall Percentage and Stability of Remission **eTable 6.** Prevalence of Remission at Each Visit **eTable 7.** Stability of Remission at Each Visit **eTable 8.** Prevalence of Remission at Each Visit – Symptoms-Only **eTable 9.** Prevalence of Remission at Each Visit – Symptoms-And-Function **eFigure 4.** Prevalence of Remission at Each Visit, Grouped by Baseline Age **eFigure 5.** Prevalence of Remission at Each Visit, Grouped by Baseline Sex **eFigure 6.** Prevalence of Remission at Each Visit, Grouped by Baseline Race **eFigure 7.** Prevalence of Remission at Each Visit, Grouped by Antipsychotic Use at That Visit **eFigure 8.** Prevalence of Remission at Each Visit, Grouped by Baseline History of Trauma **eFigure 9.** Prevalence of Remission at Each Visit, Grouped by Antidepressant Use at That Visit **eTable 10.** Comparison of Baseline Demographic and Clinical Data Between Individuals Who Remained Remitted After a Previous Remission Visit (Stable Remitters) and Individuals Who Did Not Remain Remitted After a Previous Remission Visit (Unstable Remitters). **eFigure 10.** Incidence and Prevalence of Remission at Each Visit for Different Global Assessment of Functioning (GAF) Cut-Offs **eFigure 11.** Incidence and Prevalence of Remission at Each Visit for Individuals That Did Not Use Antipsychotic Medication at Baseline **eTable 11.** Comparison of Baseline Demographic and Clinical Data Between Groups Based on the Number of Follow-Up Visits **eTable 12.** Prevalence of Remission by Site at Each Visit

Supplement 2.
Data Sharing Statement
