# Supplementary material for: Incidence, Prevalence, and Stability of Remission in Individuals With Clinical High Risk for Psychosis
Source: JAMA Netw Open. 2025 Aug 5;8(8):e2525644. doi: 10.1001/jamanetworkopen.2025.25644 (PMC12326281; doi:10.1001/jamanetworkopen.2025.25644)
Supplement: Supplement 1. — eFigure 1. Status Information Available at Each Visit eTable 1. Status Information Available at Each Visit eTable 2. Medication Information at Each Visit eTable 3. Incidence of Remission at Each Visit, Standardized by Follow-Up Time eFigure 2. Incidence and Prevalence of Remission for Each Follow-Up Visit (Non-Standardized) eTable 4. Probability of First Remission at Each Visit, Standardized by Follow-Up Time eTable 5. Status Information at the Last Recorded Visit eFigure 3. Overall Percentage and Stability of Remission eTable 6. Prevalence of Remission at Each Visit eTable 7. Stability of Remission at Each Visit eTable 8. Prevalence of Remission at Each Visit – Symptoms-Only eTable 9. Prevalence of Remission at Each Visit – Symptoms-And-Function eFigure 4. Prevalence of Remission at Each Visit, Grouped by Baseline Age eFigure 5. Prevalence of Remission at Each Visit, Grouped by Baseline Sex eFigure 6. Prevalence of Remission at Each Visit, Grouped by Baseline Race eFigure 7. Prevalence of Remission at Each Visit, Grouped by Antipsychotic Use at That Visit eFigure 8. Prevalence of Remission at Each Visit, Grouped by Baseline History of Trauma eFigure 9. Prevalence of Remission at Each Visit, Grouped by Antidepressant Use at That Visit eTable 10. Comparison of Baseline Demographic and Clinical Data Between Individuals Who Remained Remitted After a Previous Remission Visit (Stable Remitters) and Individuals Who Did Not Remain Remitted After a Previous Remission Visit (Unstable Remitters). eFigure 10. Incidence and Prevalence of Remission at Each Visit for Different Global Assessment of Functioning (GAF) Cut-Offs eFigure 11. Incidence and Prevalence of Remission at Each Visit for Individuals That Did Not Use Antipsychotic Medication at Baseline eTable 11. Comparison of Baseline Demographic and Clinical Data Between Groups Based on the Number of Follow-Up Visits eTable 12. Prevalence of Remission by Site at Each Visit [file jamanetwopen-e2525644-s001.pdf]

Supplemental Online Content

Seitz-Holland J, Jacobs GR, Reinen J, et al. Incidence, prevalence, and stability of remission in individuals with clinical high risk for psychosis. *JAMA Netw. Open*. 2025; 8(8):e2525644. doi:10.1001/jamanetworkopen.2025.25644

eFigure 1. Status Information Available at Each Visit
eTable 1. Status Information Available at Each Visit
eTable 2. Medication Information at Each Visit
eTable 3. Incidence of Remission at Each Visit, Standardized by Follow-Up Time
eFigure 2. Incidence and Prevalence of Remission for Each Follow-Up Visit (Non-Standardized)
eTable 4. Probability of First Remission at Each Visit, Standardized by Follow-Up Time
eTable 5. Status Information at the Last Recorded Visit
eFigure 3. Overall Percentage and Stability of Remission
eTable 6. Prevalence of Remission at Each Visit
eTable 7. Stability of Remission at Each Visit
eTable 8. Prevalence of Remission at Each Visit – Symptoms-Only
eTable 9. Prevalence of Remission at Each Visit – Symptoms-And-Function
eFigure 4. Prevalence of Remission at Each Visit, Grouped by Baseline Age
eFigure 5. Prevalence of Remission at Each Visit, Grouped by Baseline Sex
eFigure 6. Prevalence of Remission at Each Visit, Grouped by Baseline Race
eFigure 7. Prevalence of Remission at Each Visit, Grouped by Antipsychotic Use at That Visit
eFigure 8. Prevalence of Remission at Each Visit, Grouped by Baseline History of Trauma
eFigure 9. Prevalence of Remission at Each Visit, Grouped by Antidepressant Use at That Visit
eTable 10. Comparison of Baseline Demographic and Clinical Data Between Individuals Who Remained Remitted After a Previous Remission Visit (Stable Remitters) and Individuals Who Did Not Remain Remitted After a Previous Remission Visit (Unstable Remitters)
eFigure 10. Incidence and Prevalence of Remission at Each Visit for Different Global Assessment of Functioning (GAF) Cut-Offs
eFigure 11. Incidence and Prevalence of Remission at Each Visit for Individuals That Did Not Use Antipsychotic Medication at Baseline
eTable 11. Comparison of Baseline Demographic and Clinical Data Between Groups Based on the Number of Follow-Up Visits
eTable 12. Prevalence of Remission by Site at Each Visit

This supplemental material has been provided by the authors to give readers additional information about their work.

© 2025 Seitz-Holland J et al. *JAMA Network Open*.

eFigure 1: Status Information Available at Each Visit.

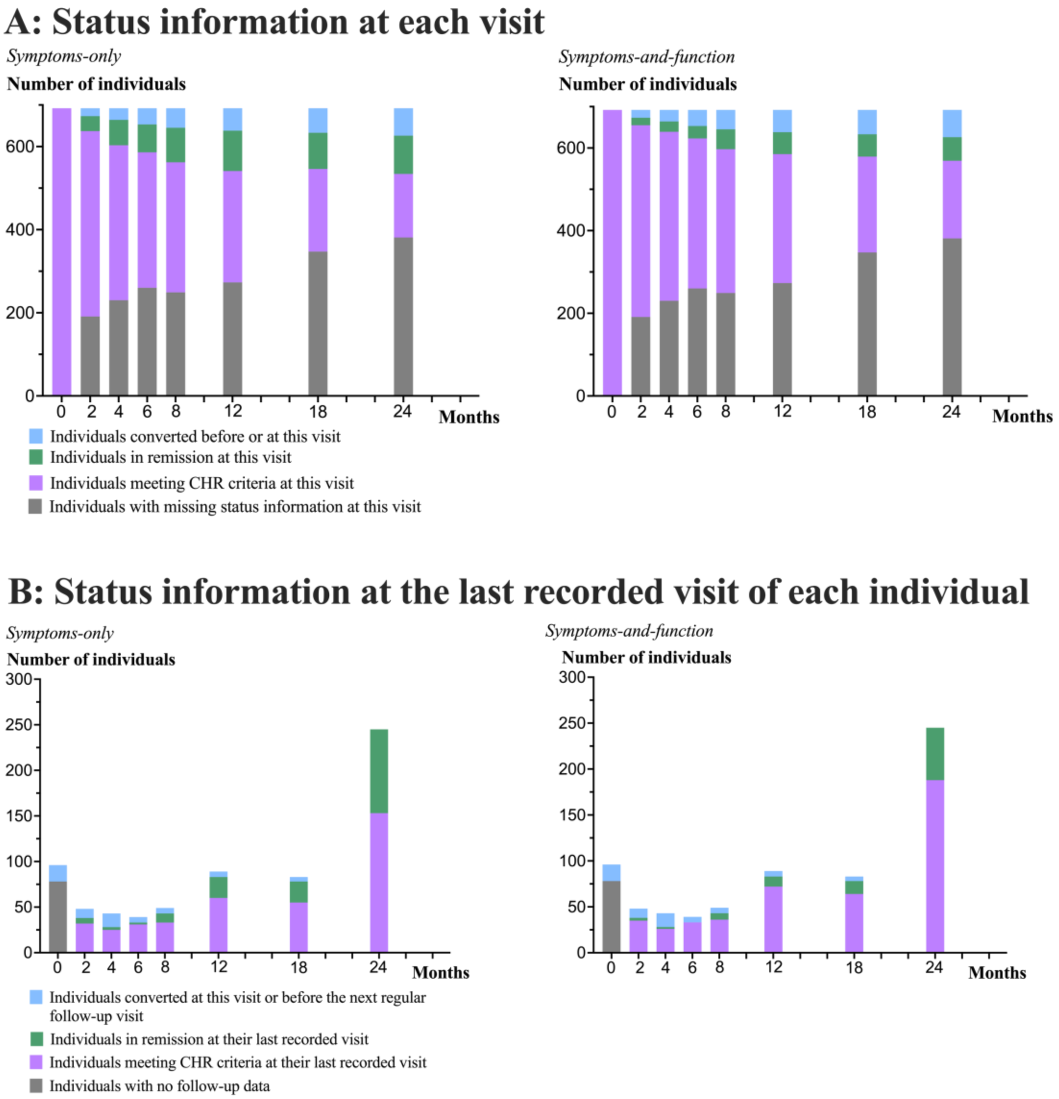

eFigure 1A presents individual status data per visit for the *symptoms-only* (left) and *symptoms-and-function* (right) remission definitions. Once an individual has converted, their conversion status remains for all visits after their conversion.

eFigure 1B shows the status information for each individual at their last recorded visit. Note that conversion visits could occur between regular follow-up visits and are displayed at the closest regular follow-up visit. Please see **Supplementary Tables 1 and 5** for more details.

© 2025 Seitz-Holland J et al. *JAMA Network Open*.

eTable 1: Status Information Available at Each Visit

	Symptoms-only				*Symptoms-and-function*			
	Individuals converted before or at this visit	Individuals in remission at this visit	Individuals meeting CHR criteria at this visit	Individuals with missing status information at this visit	Individuals converted before or at this visit	Individuals in remission at this visit	Individuals meeting CHR criteria at this visit	Individuals with missing status information at this visit
Baseline	0	0	692	0	0	0	692	0
Month 2	19	36	446	191	19	18	464	191
Month 4	28	61	373	230	28	25	409	230
Month 6	39	67	326	260	39	30	363	260
Month 8	47	83	313	249	47	48	348	249
Month 12	54	97	268	273	54	53	312	273
Month 18	59	87	199	347	59	54	232	347
Month 24	66	92	153	381	66	57	188	381

© 2025 Seitz-Holland J et al. *JAMA Network Open*.

eTable 2: Medication Information at Each Visit

		Baseline	Month 2	Month 4	Month 6	Month 8	Month 12	Month 18	Month 24
Analgesics/Opiates: n (%)		82 (12%)	25 (5%)	27 (6%)	25 (6%)	22 (6%)	26 (7%)	20 (7%)	17 (7%)
Anti-Parkinson medication: n (%)		8 (1%)	4 (1%)	5 (1%)	7 (2%)	7 (2%)	5 (1%)	3 (1%)	3 (1%)
Antidepressants: n (%)		331 (48%)	155 (32%)	157 (36%)	146 (37%)	145 (37%)	127 (35%)	98 (34%)	84 (34%)
Antiepileptic medication: n (%)		22 (3%)	11 (2%)	9 (2%)	10 (3%)	9 (2%)	10 (3%)	6 (2%)	6 (2%)
Antipsychotics: n (%)		216 (31%)	106 (22%)	94 (22%)	88 (22%)	89 (22%)	78 (21%)	59 (21%)	47 (19%)
	Unspecified antipsychotics	2 (<1%)	0	0	0	0	1 (<1%)	0	0
	Aripiprazole	103 (15%)	41 (8%)	38 (9%)	30 (8%)	37 (9%)	36 (10%)	25 (9%)	18 (7%)
	Asenapine	3 (<1%)	0	1 (<1%)	1 (<1%)	2 (1%)	1 (<1%)	1 (<1%)	1 (<1%)
	Brexpiprazole	2 (<1%)	1 (<1%)	0	0	0	0	1 (<1%)	2 (1%)
	Cariprazine	3 (<1%)	2 (<1%)	1 (<1%)	3 (1%)	2 (1%)	1 (<1%)	2 (1%)	2 (1%)
	Chlorpromazine	0	1 (<1%)	0	0	0	0	0	0
	Clozapine	2 (<1%)	0	0	1 (<1%)	1 (<1%)	0	0	0
	Fluphenazine	0	0	0	0	0	0	0	1 (<1%)
	Haloperidol	2 (<1%)	0	0	0	0	0	0	0
	Lurasidone	24 (3%)	9 (2%)	8 (2%)	7 (2%)	9 (2%)	7 (2%)	6 (2%)	3 (1%)
	Olanzapine	21 (3%)	8 (2%)	7 (2%)	6 (2%)	4 (1%)	5 (1%)	3 (1%)	3 (1%)
	Paliperidone	1 (<1%)	0	0	1 (<1%)	1 (<1%)	1 (<1%)	0	1 (<1%)
	Perphenazine	1 (<1%)	0	2 (<1%)	1 (<1%)	1 (<1%)	1 (<1%)	0	0
	Quetiapine	66 (10%)	27 (6%)	23 (5%)	24 (6%)	23 (6%)	20 (5%)	16 (6%)	13 (5%)
	Risperidone	70 (10%)	31 (6%)	19 (4%)	23 (6%)	20 (5%)	11 (3%)	10 (3%)	9 (4%)
	Ziprasidone	5 (1%)	2 (<1%)	3 (1%)	2 (1%)	2 (1%)	4 (1%)	2 (1%)	0
Anxiolytics/ Sedatives/ Hypnotics: n (%)		89 (13%)	22 (5%)	19 (4%)	15 (4%)	13 (3%)	13 (4%)	9 (3%)	10 (4%)
Mood Stabilizer: n (%)		56 (8%)	23 (5%)	19 (4%)	23 (6%)	23 (6%)	29 (8%)	16 (6%)	15 (6%)
Psychostimulants: n (%)		165 (24%)	35 (7%)	36 (8%)	30 (8%)	33 (8%)	27 (7%)	33 (12%)	33 (13%)

The numbers in eTable 2 are based on the data collected at each visit. Individuals with no medication information at this visit and individuals who converted before a visit and were not assessed at a follow-up visit are excluded.

© 2025 Seitz-Holland J et al. *JAMA Network Open*.

eTable 3: Incidence of Remission at Each Visit, Standardized by Follow-Up Time

	Symptoms-only			*Symptoms-and-function*		
	Absolute counts	Percentage	95% Confidence Interval	Absolute counts	Percentage	95% Confidence Interval
Baseline	0/692	0%		0/692	0%	
Month 2	36/484	7%	[5.1% - 9.7%]	18/484	4%	[2.0% - 5.4%]
Month 4	37/409	9%	[6.2% - 11.8%] *	14/423	3%	[1.6% - 5.0%] *
Month 6	26/344	8%	[4.8% - 10.4%]	13/373	4%	[1.6% - 5.4%]
Month 8	27/316	9%	[5.4% - 11.6%]	26/358	7%	[4.6% - 10.0%]
Month 12	(30:2)/272	6%	[2.8% - 8.2%]	(19:2)/312	3%	[1.2% - 5.2%]
Month 18	(27:3)/199	5%	[1.6% - 7.4%]	(20:3)/232	3%	[0.8% - 5.2%]
Month 24	(28:3)/156	6%	[2.1% - 9.5%]	(20:3)/190	4%	[1.0% - 6.4%]

* Indicates no overlap between the 95% Confidence Intervals for the *symptoms-only* and *symptoms-and-function* remission definitions, indicating significance.

© 2025 Seitz-Holland J et al. *JAMA Network Open*.

eFigure 2: Incidence and Prevalence of Remission for Each Follow-Up Visit (Non-Standardized)

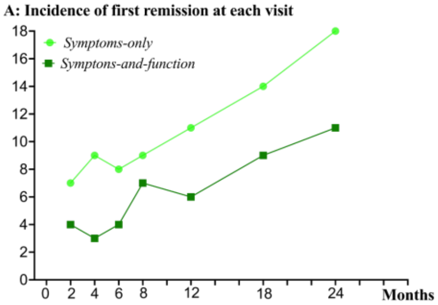

Incidence of remission at each visit

	Symptoms-only			*Symptoms-and-function*		
	Absolute counts	Percentage	95% Confidence Interval	Absolute counts	Percentage	95% Confidence Interval
Baseline	0/692	0%		0/692	0%	
Month 2	36/484	7%	[5.1% - 9.7%]	18/484	4%	[2.0% - 5.4%]
Month 4	37/409	9%	[6.2% - 11.8%] *	14/423	3%	[1.6% - 5.0%] *
Month 6	26/344	8%	[4.8% - 10.4%]	13/373	4%	[1.6% - 5.4%]
Month 8	27/316	9%	[5.4% - 11.6%]	26/358	7%	[4.6% - 10.0%]
Month 12	30/272	11%	[7.3% - 14.7%]	19/312	6%	[3.4% - 8.8%]
Month 18	27/199	14%	[8.8% - 18.4%]	20/232	9%	[5.0% - 12.2%]
Month 24	28/156	18%	[11.9% - 23.9%]	20/190	11%	[6.1% - 14.9%]

* Indicates no overlap between the 95% Confidence Intervals for the *symptoms-only* and *symptoms-and-function* remission definitions

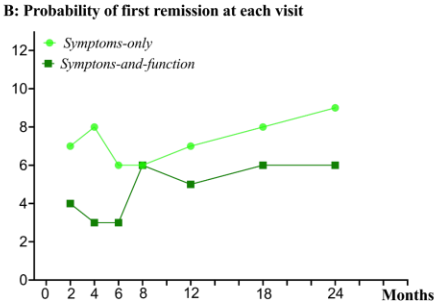

Probability of first remission at each visit

	Symptoms-only			*Symptoms-and-function*		
	Absolute counts	Percentage	95% Confidence Interval	Absolute counts	Percentage	95% Confidence Interval
Baseline	0/692	0%		0/692	0%	
Month 2	36/501	7%	[4.9% - 9.5%]	18/501	4%	[2.0% - 5.2%]
Month 4	37/462	8%	[5.5% - 10.5%] *	14/462	3%	[1.4% - 4.6%] *
Month 6	26/432	6%	[3.8% - 8.2%]	13/432	3%	[1.4% - 4.6%]
Month 8	27/443	6%	[3.9% - 8.3%]	26/443	6%	[3.7% - 8.1%]
Month 12	30/419	7%	[4.7% - 9.7%]	19/419	5%	[2.5% - 6.5%]
Month 18	27/345	8%	[5.0% - 10.6%]	20/345	6%	[3.3% - 8.3%]
Month 24	28/311	9%	[5.8% - 12.2%]	20/311	6%	[3.7% - 9.1%]

* Indicates no overlap between the 95% Confidence Intervals for the *symptoms-only* and *symptoms-and-function* remission definitions

eFigure 2 shows the incidence and probability of first remission for each follow-up visit. The tables report absolute counts, the percentage of individuals remitted relative to the individuals with data available, and 95% confidence intervals for these percentages (calculated with the Wald method).

eFigure 2A shows the incidence of first remission based on who was eligible to achieve first remission at this visit. eFigure 2B shows the probability of first remission based on everyone with available data for this visit (including converter

© 2025 Seitz-Holland J et al. *JAMA Network Open*.

eTable 4: Probability of First Remission at Each Visit, Standardized by Follow-Up Time

	Symptoms-only			*Symptoms-and-function*		
	Absolute counts	Percentage	95% Confidence Interval	Absolute counts	Percentage	95% Confidence Interval
Baseline	0/692	0%		0/692	0%	
Month 2	36/501	7%	[4.9% - 9.5%]	18/501	4%	[2.0% - 5.2%]
Month 4	37/462	8%	[5.5% - 10.5%] *	14/462	3%	[1.4% - 4.6%] *
Month 6	26/432	6%	[3.8% - 8.2%]	13/432	3%	[1.4% - 4.6%]
Month 8	27/443	6%	[3.9% - 8.3%]	26/443	6%	[3.7% - 8.1%]
Month 12	(30:2)/419	4%	[1.8% - 5.4%]	(19:2)/419	2%	[0.9% - 3.9%]
Month 18	(27:3)/345	3%	[0.9% - 4.3%]	(20:3)/345	2%	[0.5% - 3.5%]
Month 24	(28:3)/311	3%	[1.0% - 4.8%]	(20:3)/311	2%	[0.6% - 4.0%]

* Indicates no overlap between the 95% Confidence Intervals for the *symptoms-only* and *symptoms-and-function* remission definitions, indicating significance.

© 2025 Seitz-Holland J et al. *JAMA Network Open*.

eTable 5: Status Information at the Last Recorded Visit

	Symptoms-only				*Symptoms-and-function*			
	Individuals converted at this visit or before the next regular follow-up visit	Individuals in remission at their last recorded visit	Individuals meeting CHR criteria at their last recorded visit	Individuals with no follow-up data	Individuals converted at this visit or before the next regular follow-up visit	Individuals in remission at their last recorded visit	Individuals meeting CHR criteria at their last recorded visit	Individuals with no follow-up data
Baseline	18	0	0	78	18	0	0	78
Month 2	10	6	32		10	3	35	
Month 4	15	3	25		15	2	26	
Month 6	6	2	31		6	0	33	
Month 8	6	10	33		6	7	36	
Month 12	6	23	60		6	11	72	
Month 18	5	23	55		5	14	64	
Month 24	0	92	153		0	57	188	

© 2025 Seitz-Holland J et al. *JAMA Network Open*.

eFigure 3: Overall Percentage and Stability of Remission.

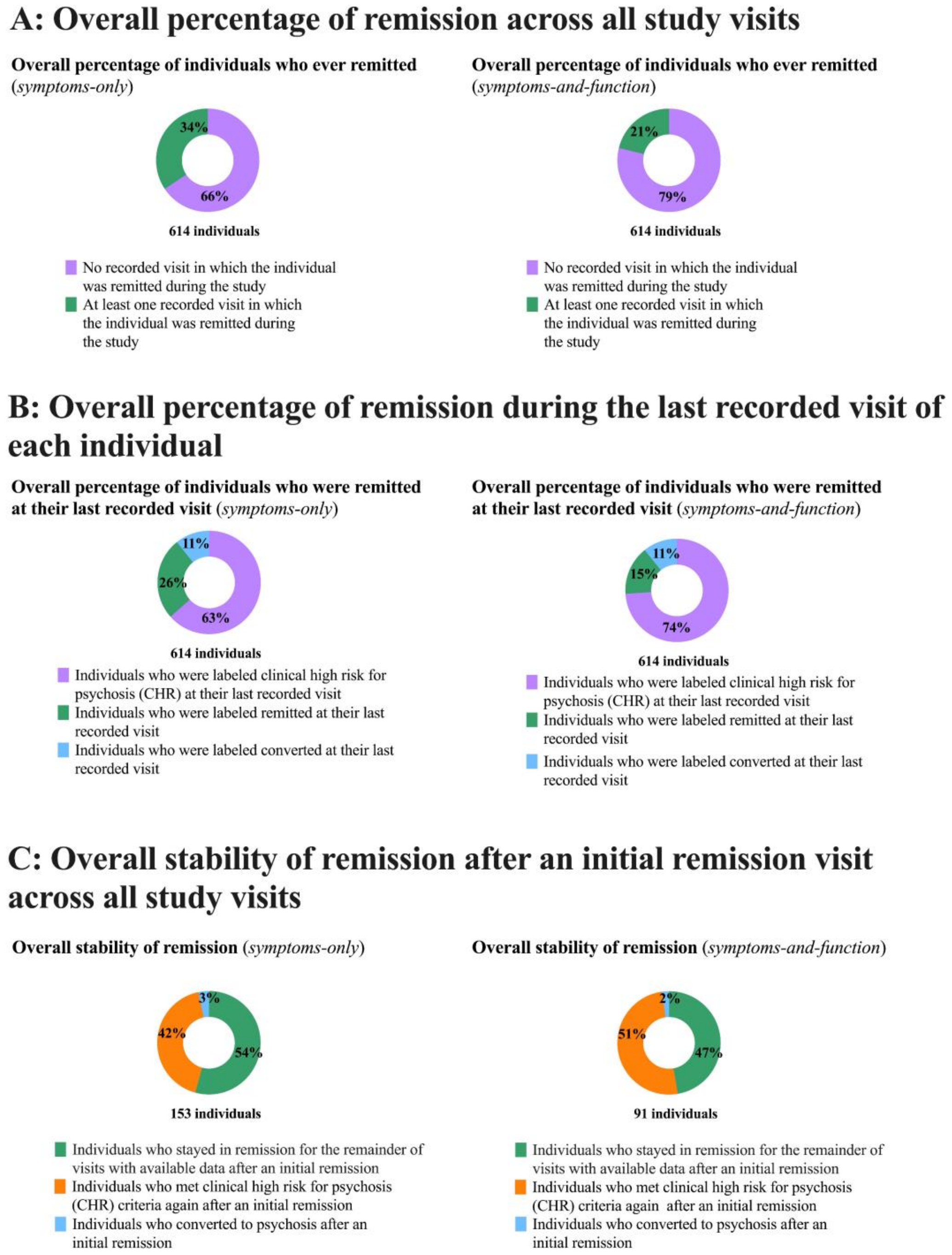

eFigure 3 shows the A) overall percentage of remission across all study visits**,** B) overall percentage of remission during the last recorded visit of each individual**,** and C) overall stability of remission after an initial remission visit across all study visits.

© 2025 Seitz-Holland J et al. *JAMA Network Open*.

eTable 6: Prevalence of Remission at Each Visit

	Symptoms-only			*Symptoms-and-function*		
	Absolute counts	Percentage	95% Confidence Interval	Absolute counts	Percentage	95% Confidence Interval
Baseline	0/692	0%		0/692	0%	
Month 2	36/501	7%	[4.9% - 9.5%]	18/501	4%	[2.0% - 5.2%]
Month 4	61/462	13%	[10.1% - 16.3%] *	25/462	5%	[3.3% - 7.5%] *
Month 6	67/432	16%	[12.1% - 18.9%] *	30/432	7%	[4.5% - 9.3%] *
Month 8	83/443	19%	[15.1% - 22.3%] *	48/443	11%	[7.9% - 13.7%] *
Month 12	97/419	23%	[19.2% - 27.2%] *	53/419	13%	[9.4% - 15.8%] *
Month 18	87/345	25%	[20.6% - 29.8%] *	54/345	16%	[11.9% - 19.5%] *
Month 24	92/311	30%	[24.5% - 34.7%] *	57/311	18%	[14.0% - 22.6%] *

* Indicates no overlap between the 95% Confidence Intervals for the *symptoms-only* and *symptoms-and-function* remission definitions, indicating significance.

© 2025 Seitz-Holland J et al. *JAMA Network Open*.

eTable 7. Stability of Remission at Each Visit

		Symptoms-only			*Symptoms-and-function*		
		Absolute counts	Percentage	95% Confidence Interval	Absolute counts	Percentage	95% Confidence Interval
Month 2	First remitter	36/36	100%		18/18	100%	
	Stable remitter						
	Unstable remitter, currently remitted						
	Unstable remitter, currently not remitted						
Month 4	First remitter	37/62	60%	[47.5% - 71.9%]	14/25	56%	[51.3% - 60.7%]
	Stable remitter	24/62	39%	[26.6% - 50.8%]	11/25	44%	[39.3% - 48.7%]
	Unstable remitter, currently remitted						
	Unstable remitter, currently not remitted	1/62	2%	[0% - 4.7%]			
Month 6	First remitter	26/77	34%	[23.2% - 44.4%]	13/34	38%	[33.4% - 43.0%]
	Stable remitter	41/77	53%	[42.1% - 64.3%]	17/34	50%	[45.1% - 54.9%]
	Unstable remitter, currently remitted						
	Unstable remitter, currently not remitted	10/77	13%	[5.5% - 20.5%]	4/34	12%	[1.0% - 22.6%]
Month 8	First remitter	27/108	25%	[16.8% - 33.2 %] *	26/64	41%	[35.8% - 45.4%] *
	Stable remitter	51/108	47%	[37.8% - 56.6%] *	20/64	31%	[26.6% - 35.8%] *
	Unstable remitter, currently remitted	5/108	5%	[0.6% - 8.6%]	2/64	3%	[0% - 7.3%]
	Unstable remitter, currently not remitted	25/108	23%	[15.2% - 31.0%]	16/64	25%	[14.4% - 35.6%]

© 2025 Seitz-Holland J et al. *JAMA Network Open*.

Month 12	First remitter	30/125	24%	[16.5% - 31.5%]	19/73	26%	[21.5% - 30.5%]
	Stable remitter	58/125	46%	[37.7% - 55.1%]	29/73	40%	[34.7% - 44.7%]
	Unstable remitter, currently remitted	9/125	7%	[2.7% - 11.7%]	5/73	7%	[1.0% - 12.6%]
	Unstable remitter, currently not remitted	28/125	22%	[15.1% - 29.7%]	20/73	27%	[17.2% - 37.6%]
Month 18	First remitter	27/119	23%	[15.2% - 30.2%]	20/76	26%	[21.2% - 31.4%]
	Stable remitter	50/119	42%	[33.1% - 50.9%]	27/76	36%	[30.0% - 41.0%]
	Unstable remitter, currently remitted	10/119	8%	[3.4% - 13.4%]	7/76	9%	[2.7% - 15.7%]
	Unstable remitter, currently not remitted	32/119	27%	[18.9% - 34.9%]	22/76	29%	[18.7% - 39.1%]
Month 24	First remitter	28/122	23%	[15.5% - 30.5%]	20/77	26%	[20.5% - 31.5%]
	Stable remitter	49/122	40%	[31.5% - 48.9%]	29/77	38%	[31.6% - 43.8%]
	Unstable remitter, currently remitted	15/122	12%	[6.5% - 18.1%]	8/77	10%	[3.6% - 17.2%]
	Unstable remitter, currently not remitted	30/122	25%	[17.0% - 32.2%]	20/77	26%	[16.2% - 35.8%]

* Indicates no overlap between the 95% Confidence Intervals for the *symptoms-only* and *symptoms-and-function* remission definitions, indicating significance.

© 2025 Seitz-Holland J et al. *JAMA Network Open*.

eTable 8: Prevalence of Remission at Each Visit – *Symptoms-Only*

	No previous remission visit			One previous remission visit			Two previous remission visits			More than two previous remission visits		
	Absolute counts	Percentage	95% Confidence Interval	Absolute counts	Percentage	95% Confidence Interval	Absolute counts	Percentage	95% Confidence Interval	Absolute counts	Percentage	95% Confidence Interval
Baseline	0/692	0%										
Month 2	36/501	7%	[4.9% - 9.4%]									
Month 4	37/437	9%	[5.9% - 11.1%]	24/25	96%	[88.3% - 100%]						
Month 6	26/381	7%	[4.3% - 9.4%]	21/30	70%	[53.6% - 86.4%]	20/21	95%	[86.1% - 100%]			
Month 8	27/362	8%	[4.8% - 10.2%]	21/41	51%	[35.9% - 66.5%]	18/23	78%	[61.4% - 95.1%]	17/17	100%	
Month 12	30/324	9%	[6.1% - 12.4%]	22/40	55%	[39.6% - 70.4%]	14/21	67%	[46.5% - 86.8%]	31/34	91%	[81.6% - 100%]
Month 18	27/253	11%	[6.9% - 14.5%]	16/34	47%	[30.3% - 63.8%]	15/24	63%	[43.1% - 81.9%]	29/34	85%	[73.4% - 97.2%]
Month 24	28/217	13%	[8.4% - 17.4%]	19/37	51%	[35.2% – 67.5%]	16/20	80%	[62.5% - 97.5%]	29/37	78%	[65.1% - 91.6%]

© 2025 Seitz-Holland J et al. *JAMA Network Open*.

eTable 9: Prevalence of remission at each visit – *symptoms-and-function*

	No previous remission visit			One previous remission visit			Two previous remission visits			More than two previous remission visits		
	Absolute counts	Percentage	95% Confidence Interval	Absolute counts	Percentage	95% Confidence Interval	Absolute counts	Percentage	95% Confidence Interval	Absolute counts	Percentage	95% Confidence Interval
Baseline	0/692	0%										
Month 2	18/501	4%	[2.0% - 5.2%]									
Month 4	14/451	3%	[1.5% - 4.7%]	11/11	100%							
Month 6	13/411	3%	[1.5% - 4.9%]	8/12	67%	[40.0% - 93.3%]	9/9	100%				
Month 8	26/405	6%	[4.0% - 8.8%]	6/20	30%	[9.9% - 50.1%]	7/9	78%	[50.6% - 100%]	9/9	100%	
Month 12	19/365	5%	[2.9% - 7.5%]	17/32	53%	[35.8% - 70.4%]	5/7	71%	[38.0% - 100%]	12/15	80%	[59.8% - 100%]
Month 18	20/289	7%	[4.0% - 9.8%]	10/26	39%	[19.8% - 57.2%]	11/16	69%	[46.0% - 91.5%]	13/14	93%	[79.4% - 100%]
Month 24	20/254	8%	[4.6% - 11.2%]	13/29	45%	[26.7% - 62.9%]	9/11	82%	[59.0% - 100%]	15/17	88%	[72.9% - 100%]

© 2025 Seitz-Holland J et al. *JAMA Network Open*.

eFigure 4: Prevalence of Remission at Each Visit, Grouped by Baseline Age.

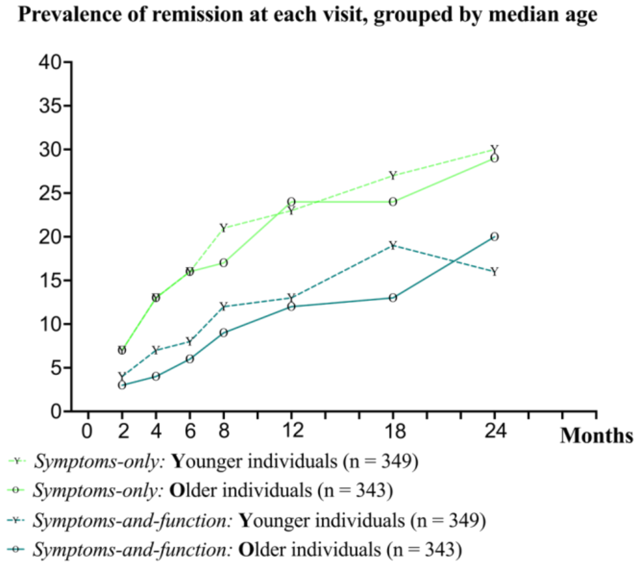

Prevalence of remission at each visit – *symptoms-only*

	Younger			Older		
	Absolute counts	Percentage	95% Confidence Interval	Absolute counts	Percentage	95% Confidence Interval
Baseline	0/349	0%		0/343	0%	
Month 2	18/253	7%	[3.9% - 10.3%]	18/248	7%	[4.1% - 10.5%]
Month 4	30/230	13%	[8.7% - 17.3%]	31/232	13%	[9.0% - 17.8%]
Month 6	34/220	16%	[10.7% - 20.3%]	33/212	16%	[10.7% - 20.5%]
Month 8	45/220	21%	[15.2% - 25.8%]	38/223	17%	[12.1% - 21.9%]
Month 12	46/204	23%	[16.8% - 28.2%]	51/215	24%	[18.0% - 29.4%]
Month 18	43/162	27%	[19.7% - 33.3%]	44/183	24%	[17.8% - 30.2%]
Month 24	43/144	30%	[22.4% - 37.4%]	49/167	29%	[22.4% - 36.2%]

* Indicates no overlap between the 95% Confidence Intervals for the younger and older age groups

Prevalence of remission at each visit – *symptoms-and-function*

	Younger			Older		
	Absolute counts	Percentage	95% Confidence Interval	Absolute counts	Percentage	95% Confidence Interval
Baseline	0/349	0%		0/343	0%	
Month 2	11/253	4%	[1.8% - 6.8%]	7/248	3%	[0.7% - 4.9%]
Month 4	15/230	7%	[3.3% - 9.7%]	10/232	4%	[1.7% - 6.9%]
Month 6	18/220	8%	[4.6% - 11.8%]	12/212	6%	[2.6% - 8.8%]
Month 8	27/220	12%	[8.0% - 16.6%]	21/223	9%	[5.6% - 13.2%]
Month 12	27/204	13%	[8.6% - 17.8%]	26/215	12%	[7.7% - 16.5%]
Month 18	30/162	19%	[12.5% - 24.5%]	24/183	13%	[8.2% - 18.0%]
Month 24	23/144	16%	[10.0% - 22.0%]	34/167	20%	[14.3% - 26.5%]

* Indicates no overlap between the 95% Confidence Intervals for the younger and older age groups

eFigure 4 shows the prevalence of remission for the *symptoms-only* and *symptoms-and-function* definitions divided by age group, as assessed at baseline. Individuals were split based on the median age (<= 17.9 and > 17.9 years) at baseline in a younger (n= 349) and older group (n= 343).

The tables report absolute counts, the percentage of individuals remitted relative to the individuals with data available, and 95% confidence intervals for these percentages (calculated with the Wald method).

Individuals who converted at or before a visit were included in the analyses.

© 2025 Seitz-Holland J et al. *JAMA Network Open*.

eFigure 5: Prevalence of Remission at Each Visit, Grouped by Baseline Sex.

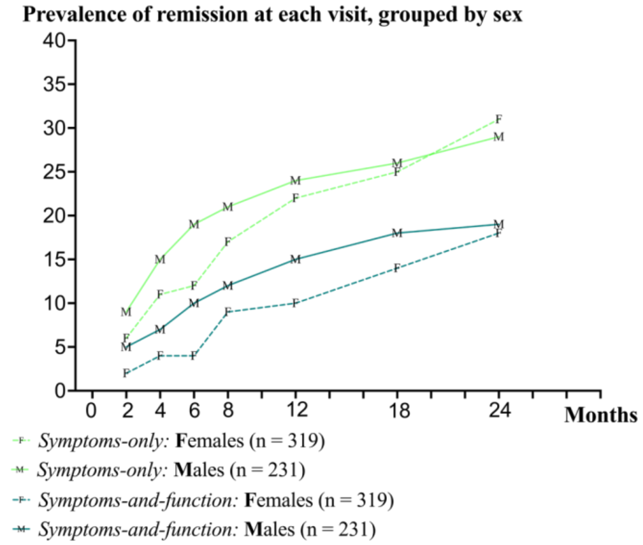

Prevalence of remission at each visit – *symptoms-only*

	Females			Males		
	Absolute counts	Percentage	95% Confidence Interval	Absolute counts	Percentage	95% Confidence Interval
Baseline	0/319	0%		0/373	0%	
Month 2	13/238	6%	[2.6% - 8.4%]	23/263	9%	[5.3% - 12.1%]
Month 4	24/213	11%	[7.0% - 15.6%]	37/249	15%	[10.5% - 19.3%]
Month 6	23/196	12%	[7.2% - 16.2%]	44/236	19%	[13.6% - 23.6%]
Month 8	34/205	17%	[11.5% - 21.7%]	49/238	21%	[15.5% - 25.7%]
Month 12	42/192	22%	[16.1% - 27.7%]	55/227	24%	[18.6% - 29.8%]
Month 18	40/162	25%	[18.1% - 31.3%]	47/183	26%	[19.4% - 32.0%]
Month 24	45/147	31%	[23.2% - 38.0%]	47/164	29%	[21.8% - 35.6%]

* Indicates no overlap between the 95% Confidence Intervals for females and males

Prevalence of remission at each visit – *symptoms-and-function*

	Females			Males		
	Absolute counts	Percentage	95% Confidence Interval	Absolute counts	Percentage	95% Confidence Interval
Baseline	0/319	0%		0/373	0%	
Month 2	4/238	2%	[0.1% - 3.3%]	14/263	5%	[2.6% - 8.0%]
Month 4	8/213	4%	[1.2% - 6.4%]	17/249	7%	[3.7% - 9.9%]
Month 6	7/196	4%	[1.0% - 6.2%]	23/236	10%	[5.9% - 13.5%]
Month 8	19/205	9%	[5.3% - 13.3%]	29/238	12%	[8.0% - 16.4%]
Month 12	20/192	10%	[6.1% - 14.7%]	33/227	15%	[9.9% - 19.1%]
Month 18	22/162	14%	[8.3% - 18.9%]	32/183	18%	[12.0% - 23.0%]
Month 24	26/147	18%	[11.5% - 23.9%]	31/164	19%	[12.9% - 24.9%]

* Indicates no overlap between the 95% Confidence Intervals for females and males

eFigure 5 shows the prevalence of remission for the *symptoms-only* and *symptoms-and-function* definitions divided by sex, as assessed at baseline. Individuals were split into females (n=319) and males (n=231).

The tables report absolute counts, the percentage of individuals remitted relative to the individuals with data available, and 95% confidence intervals for these percentages (calculated with the Wald method).

Individuals who converted at or before a visit were included in the analyses.

© 2025 Seitz-Holland J et al. *JAMA Network Open*.

eFigure 6: Prevalence of Remission at Each Visit, Grouped by Baseline Race.

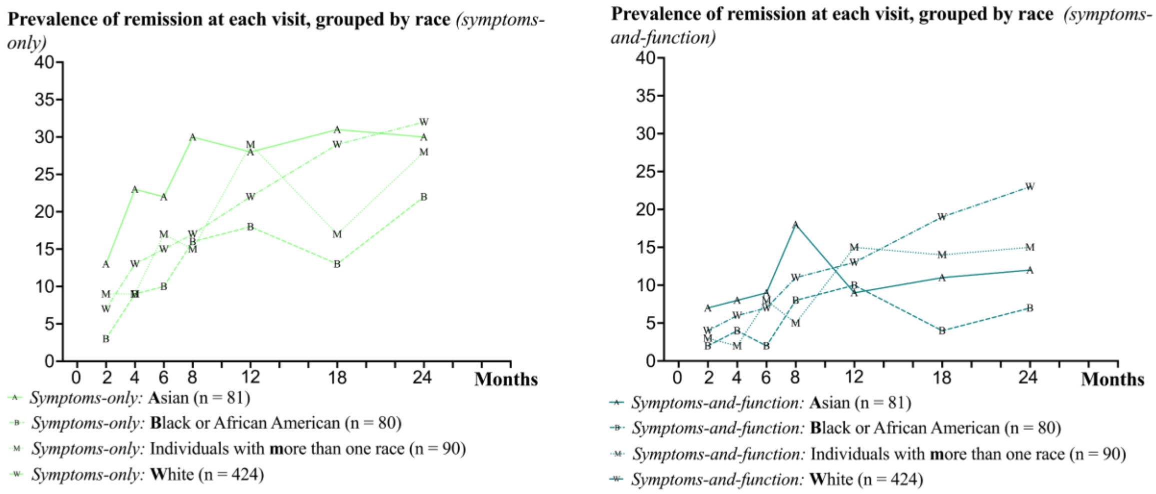

Prevalence of remission at each visit – *symptoms-only*

	Asian			Black or African American			More than one race			White		
	Absolute counts	Percentage	95% Confidence Interval	Absolute counts	Percentage	95% Confidence Interval	Absolute counts	Percentage	95% Confidence Interval	Absolute counts	Percentage	95% Confidence Interval
Baseline	0/81	0%		0/80	0%		0/90	0%		0/424	0%	
Month 2	7/54	13%	[4.0% - 22.0%]	2/61	3%	[0% - 7.8%]	6/64	9%	[2.3% - 16.5%]	21/307	7%	[4.0% - 9.6%]
Month 4	11/48	23%	[11.0% - 34.8%]	5/53	9%	[1.5% - 17.3%]	5/59	9%	[1.4% - 15.6%]	39/290	13%	[9.5% - 17.3%]
Month 6	10/46	22%	[9.8% - 33.6%]	5/51	10%	[1.6% - 18.0%]	10/60	17%	[7.3% - 26.1%]	39/262	15%	[10.6% - 19.2%]
Month 8	15/50	30%	[17.3% - 42.7%]	8/51	16%	[5.7% - 25.7%]	10/65	15%	[6.6% - 24.2%]	46/265	17%	[12.8% - 22.0%]
Month 12	12/43	28%	[14.5% - 41.3%]	9/50	18%	[7.4% - 28.6%]	16/55	29%	[17.1% - 41.1%]	57/259	22%	[17.0% - 27.0%]
Month 18	11/36	31%	[15.5% - 45.7%]	6/45	13%	[3.4% - 23.2%]	7/42	17%	[5.4% - 28.0%]	61/211	29%	[22.8% - 35.0%]
Month 24	10/33	30%	[14.6% - 46.0%]	9/41	22%	[9.3% - 34.7%]	13/46	28%	[15.3% - 41.3%]	59/184	32%	[25.4% - 38.8%]

Prevalence of remission at each visit – *symptoms-and-function*

	Asian			Black or African American			More than one race			White		
	Absolute counts	Percentage	95% Confidence Interval	Absolute counts	Percentage	95% Confidence Interval	Absolute counts	Percentage	95% Confidence Interval	Absolute counts	Percentage	95% Confidence Interval
Baseline	0/81	0%		0/80	0%		0/90	0%		0/424	0%	
Month 2	4/54	7%	[0.4% - 14.4%]	1/61	2%	[0% - 4.7%]	2/64	3%	[0% - 7.3%]	11/307	4%	[1.5% - 5.7%]
Month 4	4/48	8%	[0.5% - 16.1%]	2/53	4%	[0% - 8.9%]	1/59	2%	[0% - 5.0%]	17/290	6%	[3.2% - 8.6%]
Month 6	4/46	9%	[0.6% - 16.8%]	1/51	2%	[0% - 5.8%]	5/60	8%	[1.3% - 15.3%]	19/262	7%	[4.2% - 10.4%]
Month 8	9/50	18%	[7.4% - 28.6%]	4/51	8%	[0.4% - 15.2%]	3/65	5%	[0% - 9.7%]	29/265	11%	[7.1% - 14.7%]
Month 12	4/43	9%	[0.6% - 18.0%]	5/50	10%	[1.7% - 18.3%]	8/55	15%	[5.2% - 23.8%]	34/259	13%	[9.0% - 17.2%]
Month 18	4/36	11%	[0.8% - 21.4%]	2/45	4%	[0% - 10.4%]	6/42	14%	[3.7% - 24.9%]	40/211	19%	[13.7% - 24.3%]
Month 24	4/33	12%	[1.0% - 23.2%]	3/41	7%	[0% - 15.3%]	7/46	15%	[4.8% - 25.6%]	42/184	23%	[16.7% - 28.9%]

eFigure 6 shows the prevalence of remission for the *symptoms-only* and *symptoms-and-function* definitions divided by race, as assessed at baseline. Individuals were split into Asian (n=81), Black or African American (n=80), individuals with more than one race (n=90), and White (n=424) groups. The tables report absolute counts, the percentage of individuals remitted relative to the individuals with data available, and 95% confidence intervals for these percentages (calculated with the Wald method). Individuals who converted at or before a visit were included in the analyses.

© 2025 Seitz-Holland J et al. *JAMA Network Open*.

eFigure 7: Prevalence of Remission at Each Visit, Grouped by Antipsychotic Use at That Visit.

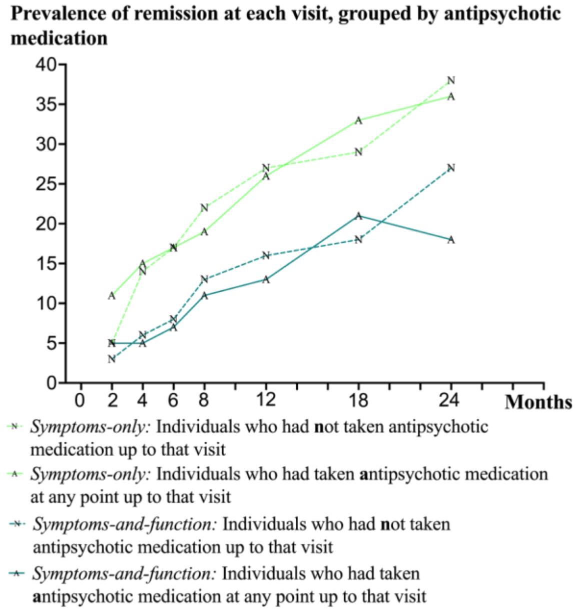

Prevalence of remission at each visit – *symptoms-only*

	No antipsychotic medication			Antipsychotic medication		
	Absolute counts	Percentage	95% Confidence Interval	Absolute counts	Percentage	95% Confidence Interval
Month 2	17/317	5%	[2.9% - 7.9%]	19/167	11%	[6.6% - 16.2%]
Month 4	39/282	14%	[9.8% - 17.8%]	22/152	15%	[8.9% - 20.1%]
Month 6	43/256	17%	[12.2% - 21.4%]	24/138	17%	[11.1% - 23.7%]
Month 8	56/256	22%	[16.8% - 27.0%]	27/140	19%	[12.8% - 25.8%]
Month 12	60/222	27%	[21.2% - 32.8%]	37/143	26%	[18.7% - 33.1%]
Month 18	53/182	29%	[22.5% - 35.7%]	34/104	33%	[23.7% - 41.7%]
Month 24	58/151	38%	[30.6% - 46.2%]	34/94	36%	[26.5% - 45.9%]

* Indicates no overlap between the 95% Confidence Intervals for individuals on no antipsychotic medication and individuals on antipsychotic medication

Prevalence of remission at each visit – *symptoms-and-function*

	No antipsychotic medication			Antipsychotic medication		
	Absolute counts	Percentage	95% Confidence Interval	Absolute counts	Percentage	95% Confidence Interval
Month 2	10/317	3%	[1.3% - 5.1%]	8/167	5%	[1.6% - 8.0%]
Month 4	17/282	6%	[3.2% - 8.8%]	8/152	5%	[1.7% - 8.9%]
Month 6	20/256	8%	[4.5% - 11.1%]	10/138	7%	[2.9% - 11.5%]
Month 8	32/256	13%	[8.4% - 16.6%]	16/140	11%	[6.1% - 16.7%]
Month 12	35/222	16%	[11.0% - 20.6%]	18/143	13%	[7.2% - 18.0%]
Month 18	32/182	18%	[12.1% - 23.1%]	22/104	21%	[13.3% - 29.1%]
Month 24	40/151	27%	[19.5% - 33.5%]	17/94	18%	[10.3% - 25.9%]

* Indicates no overlap between the 95% Confidence Intervals for individuals on no antipsychotic medication and individuals on antipsychotic medication

eFigure 7 shows the prevalence of remission for the *symptoms-only* and *symptoms-and-function* definitions divided by antipsychotic use. For each visit, we calculated the prevalence of remission for individuals who used antipsychotic medication at or before that visit and individuals who had not used antipsychotic medication at or before that visit.

The tables report absolute counts, the percentage of individuals remitted relative to the individuals with data available, and 95% confidence intervals for these percentages (calculated with the Wald method).

Please note that information about antipsychotic use was not available for all individuals and that the converters were excluded from the present analyses, given that their antipsychotic use was not reported after conversion.

© 2025 Seitz-Holland J et al. *JAMA Network Open*.

eFigure 8: Prevalence of Remission at Each Visit, Grouped by Baseline History of Trauma

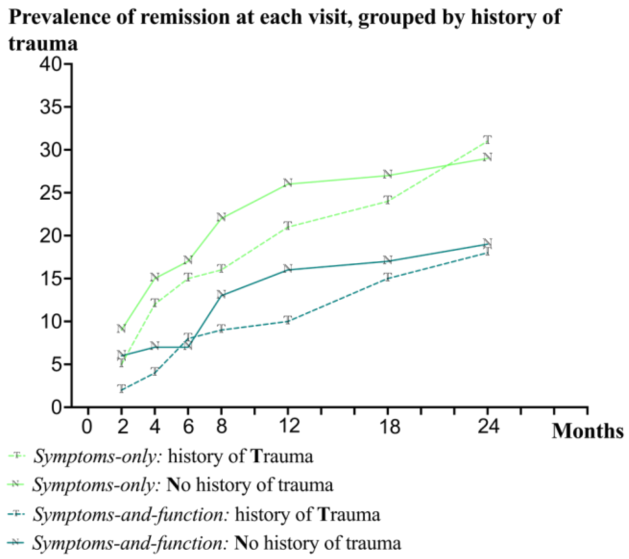

Prevalence of remission at each visit – *symptoms-only*

	Trauma			No Trauma		
	Absolute counts	Percentage	95% Confidence Interval	Absolute counts	Percentage	95% Confidence Interval
Baseline	0/342	0%		0/339	0%	
Month 2	13/240	5%	[2.5% - 8.3%]	23/256	9%	[5.5% - 12.5%]
Month 4	27/230	12%	[7.5% - 15.9%]	34/226	15%	[10.3% - 19.7%]
Month 6	31/212	15%	[9.8% - 19.4%]	36/215	17%	[11.7% - 21.7%]
Month 8	35/218	16%	[11.2% - 21.0%]	48/221	22%	[16.3% - 27.1%]
Month 12	43/210	21%	[15.0% - 26.0%]	54/205	26%	[20.3% - 32.3%]
Month 18	42/172	24%	[18.0% - 30.8%]	45/168	27%	[20.1% - 33.5%]
Month 24	49/160	31%	[23.5% - 37.7%]	43/147	29%	[21.9% - 36.7%]

* Indicates no overlap between the 95% Confidence Intervals for the younger and older age groups

Prevalence of remission at each visit – *symptoms-and-function*

	Trauma			No Trauma		
	Absolute counts	Percentage	95% Confidence Interval	Absolute counts	Percentage	95% Confidence Interval
Baseline	0/342	0%		0/339	0%	
Month 2	4/240	2%	[0.1% - 3.3%]	14/256	6%	[2.7% - 8.3%]
Month 4	10/230	4%	[1.7% - 6.9%]	15/226	7%	[3.4% - 9.8%]
Month 6	16/212	8%	[4.0% - 11.0%]	14/215	7%	[3.2% - 9.8%]
Month 8	19/218	9%	[5.0% - 12.4%]	29/221	13%	[8.7% - 17.5%]
Month 12	20/210	10%	[5.5% - 13.5%]	33/205	16%	[11.1% - 21.1%]
Month 18	25/172	15%	[9.2% - 19.8%]	29/168	17%	[11.6% - 23.0%]
Month 24	29/160	18%	[12.1% - 24.1%]	28/147	19%	[12.7% - 25.3%]

* Indicates no overlap between the 95% Confidence Intervals for the younger and older age groups

eFigure 8 shows the prevalence of remission for the *symptoms-only* and *symptoms-and-function* definitions divided by history of trauma. Individuals were split into individuals who reported a previous trauma at baseline (n = 342) and individuals who did not report a previous trauma (n = 339) as determined by the Childhood Trauma and Abuse scale.

The tables report absolute counts, the percentage of individuals remitted relative to the individuals with data available, and 95% confidence intervals for these percentages (calculated with the Wald method).

Individuals who converted at or before a visit were included in the analyses.

© 2025 Seitz-Holland J et al. *JAMA Network Open*.

eFigure 9: Prevalence of Remission at Each Visit, Grouped by Antidepressant Use at That Visit.

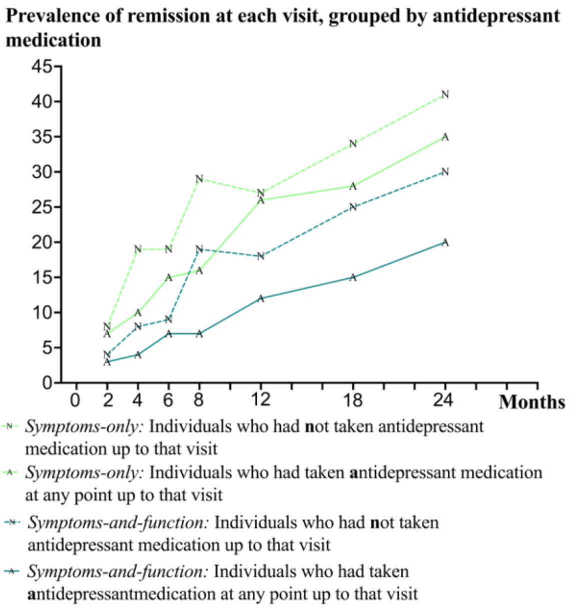

Prevalence of remission at each visit – *symptoms-only*

	No antidepressant medication			Antidepressant medication		
	Absolute counts	Percentage	95% Confidence Interval	Absolute counts	Percentage	95% Confidence Interval
Month 2	18/230	8%	[4.3% - 11.3%]	18/254	7%	[3.9% - 10.3%]
Month 4	37/200	19%	[13.1% - 23.9%]	24/234	10%	[6.4% - 14.2%]
Month 6	33/172	19%	[13.3% - 25.1%]	34/222	15%	[10.6% - 20.1%]
Month 8	47/165	29%	[21.6% - 35.4%]*	36/231	16%	[10.9% - 20.3%]*
Month 12	40/148	27%	[19.8% - 34.2%]	57/217	26%	[20.4% - 32.2%]
Month 18	39/114	34%	[25.5% - 42.9%]	48/172	28%	[21.2% - 34.6%]
Month 24	36/87	41%	[31.1% - 51.7%]	56/158	35%	[27.9% - 42.9%]

* Indicates no overlap between the 95% Confidence Intervals for individuals on no antipsychotic medication and individuals on antipsychotic medication

Prevalence of remission at each visit – *symptoms-and-function*

	No antidepressant medication			Antidepressant medication		
	Absolute counts	Percentage	95% Confidence Interval	Absolute counts	Percentage	95% Confidence Interval
Month 2	10/230	4%	[1.7% - 6.9%]	8/254	3%	[1.0% - 5.2%]
Month 4	15/200	8%	[3.8% - 11.2%]	10/234	4%	[1.7% - 6.9%]
Month 6	15/172	9%	[4.5% - 12.9%]	15/222	7%	[3.5% - 10.1%]
Month 8	32/165	19%	[13.4% - 25.4%]*	16/231	7%	[3.6% - 10.2%]*
Month 12	26/148	18%	[11.5% - 23.7%]	27/217	12%	[8.0% - 16.8%]
Month 18	28/114	25%	[16.7% - 32.5%]	26/172	15%	[9.7% - 20.5%]
Month 24	26/87	30%	[20.3% - 39.5%]	31/158	20%	[13.4% - 25.8%]

* Indicates no overlap between the 95% Confidence Intervals for individuals on no antipsychotic medication and individuals on antipsychotic medication

eFigure 9 shows the prevalence of remission for the *symptoms-only* and *symptoms-and-function* definitions divided by antidepressant use. For each visit, we calculated the prevalence of remission for individuals who used antidepressant medication at or before that visit and individuals who had not used antidepressant medication at or before that visit.

The tables report absolute counts, the percentage of individuals remitted relative to the individuals with data available, and 95% confidence intervals for these percentages (calculated with the Wald method).

Please note that information about antidepressant use was not available for all individuals and that the converters were excluded from the present analyses, given that their antidepressant use was not reported after conversion.

© 2025 Seitz-Holland J et al. *JAMA Network Open*.

eTable 10: Comparison of Baseline Demographic and Clinical Data Between Individuals Who Remained Remitted After a Previous Remission Visit (Stable Remitters) and Individuals Who Did Not Remain Remitted After a Previous Remission Visit (Unstable Remitters).

		Symptoms-only			*Symptoms-and-function*		
		Unstable remitters (n = 70)	Stable remitters (n = 83)	Test statistic	Unstable remitters (n = 48)	Stable remitters (n = 43)	Test statistic
Age (years)	Mean (std)	20.2 (4.5)	19.1 (3.9)	OR: 0.94 [0.87, 1.02], p=0.14	19.9 (4.6)	19 (3.6)	OR: 0.95 [0.86, 1.06], p=0.37
Sex	Female: n (%)	33 (47%)	32 (39%)	OR: 1.42 [0.75, 2.72], p=0.28	25 (52%)	15 (35%)	OR: 2.03 [0.88, 4.8], p=0.1
Race	Asian: n (%)	10 (14%)	15 (18%)	OR: 1.26 [0.52, 3.18], p=0.61	6 (12%)	6 (14%)	OR: 0.94 [0.26, 3.31], p=0.92
	Black or African American: n (%)	7 (10%)	5 (6%)	OR: 0.6 [0.17, 2.02], p=0.41	5 (10%)	1 (2%)	OR: 0.19 [0.01, 1.25], p=0.14
	More than one race: n (%)	9 (13%)	9 (11%)	OR: 0.84 [0.3, 2.34], p=0.74	7 (15%)	4 (9%)	OR: 0.54 [0.13, 1.96], p=0.36
	White: n (%)	43 (61%)	51 (61%)		29 (60%)	31 (72%)	
Antipsychotic use	n (%)	14 (20%)	28 (34%)	OR: 2.04 [0.98, 4.37], p=0.06	12 (25%)	13 (30%)	OR: 1.3 [0.52, 3.3], p=0.58
Antidepressant use	n (%)	31 (44%)	31 (37%)	OR: 0.75 [0.39, 1.43], p=0.38	19 (40%)	13 (30%)	OR: 0.66 [0.27, 1.57], p=0.35
Trauma	Trauma occurrence: n (%)	39 (56%)	34 (41%)	OR: 0.55 [0.29, 1.05], p=0.07	27 (56%)	17 (41%)	OR: 0.51 [0.22, 1.17], p=0.11
	Number of traumas: mean (std)	1.0 (1.2)	0.9 (1.2)	OR: 0.89 [0.68, 1.17], p=0.41	1.0 (1.1)	0.9 (1.3)	OR: 0.89 [0.63, 1.26], p=0.53
	Trauma impact: mean (std)	3.6 (4.2)	3.1 (4.8)	OR: 0.98 [0.91, 1.05], p=0.48	3.7 (4.7)	3.0 (4.5)	OR: 0.97 [0.88, 1.06], p=0.48

© 2025 Seitz-Holland J et al. *JAMA Network Open*.

MATRICS: mean (std)	Hopkins Verbal Learning Test-Revised	27.3 (5.5)	27.1 (5.1)	OR: 1 [0.93, 1.06], p=0.9	27.3 (4.9)	27.0 (4.8)	OR: 1.07 [0.96, 1.19], p=0.21
	Brief Assessment of Cognition in Schizophrenia	60.7 (15.2)	62.6 (13.4)	OR: 1.03 [1, 1.07], p=0.03*	61.0 (13.4)	64.1 (14.4)	OR: 1.04 [1, 1.09], p=0.06
	Letter-number-span	15.5 (3.6)	14.7 (3.5)	OR: 0.94 [0.85, 1.04], p=0.26	15.2 (3.5)	15.0 (3.1)	OR: 0.95 [0.83, 1.1], p=0.52
Wechsler Abbreviated Scale of Intelligence: mean (std)	Matrix score	21.5 (4.6)	20.7 (3.8)	OR: 0.96 [0.88, 1.03], p=0.25	21.0 (4.2)	20.8 (3.7)	OR: 0.99 [0.89, 1.1], p=0.82
	Vocabulary score	38.4 (5.8)	38.5 (6.5)	OR: 1 [0.95, 1.06], p=0.94	38.1 (5.8)	38.5 (6.1)	OR: 1.01 [0.94, 1.09], p=0.8
GAF: mean (std)	Current score	57.1 (11.5)	61.4 (14.2)	OR: 1 [0.98, 1.04], p=0.74	61.5 (11.4)	68.8 (13.1)	OR: 1.03 [0.99, 1.07], p=0.18
	Highest score in the last	59.4 (12.2)	59 (14.0)	OR: 0.99 [0.96, 1.01], p=0.32	61 (13.3)	64.2 (14.3)	OR: 1.02 [0.99, 1.05], p=0.24
SIPS: mean (std)	Positive	7.3 (4.0)	5.7 (4.8)	OR: 1 [0.9, 1.09], p=0.92	6.8 (4.2)	5.4 (4.8)	OR: 0.99 [0.88, 1.12], p=0.9
	Negative	9.4 (6.4)	7.3 (6.4)	OR: 0.99 [0.94, 1.04], p=0.62	7.3 (5.2)	4.9 (5.2)	OR: 0.96 [0.9, 1.03], p=0.3
	Disorganized	3.5 (2.9)	2.6 (2.7)	OR: 0.99 [0.89, 1.1], p=0.79	2.7 (2.0)	2.1 (2.6)	OR: 1.04 [0.89, 1.22], p=0.61
	General	6.8 (4.1)	5.2 (4.3)	OR: 0.95 [0.88, 1.03], p=0.24	6.1 (3.6)	3.6 (3.7)	OR: 0.94 [0.84, 1.04], p=0.22

Abbreviations: GAF = Global Assessment of Functioning; SIPS = Structured Interview for Psychosis-risk Syndromes; std = standard deviation
Supplementary Table 3 is based on baseline data for individuals who had at least one follow-up visit after a previous remission visit. Please note that individuals who converted after a previous remission were included as unstable remitters, and not all individuals had information for all variables. * Represents statistically significant group differences (not corrected for multiple comparisons). ** Represents statistically significant group differences after correction for multiple comparisons using false discovery rate.

© 2025 Seitz-Holland J et al. *JAMA Network Open*.

eFigure 10: Incidence and Prevalence of Remission at Each Visit for Different Global Assessment of Functioning (GAF) Cut-Offs.

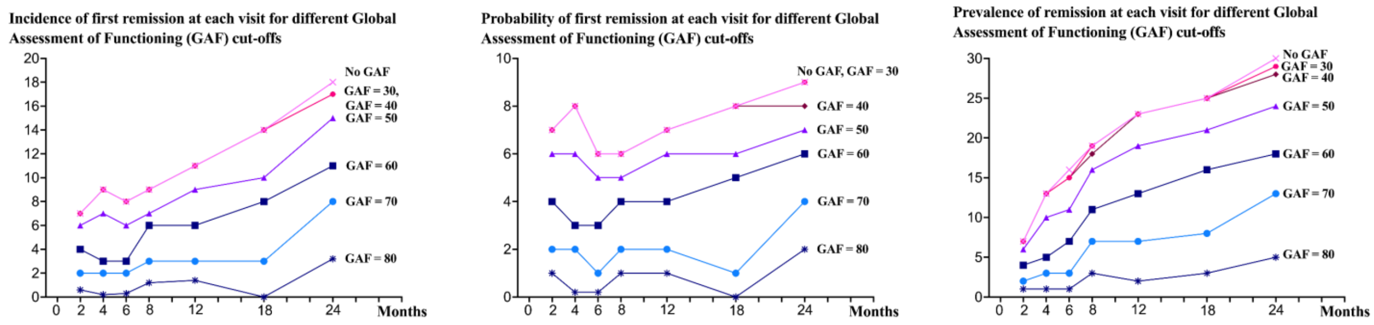

eFigure 10 shows the incidence, probability of first remission, and prevalence of remission for the *symptoms-and-function* definition using different GAF cut-offs. As expected, higher cut-offs are related to lower remission incidence, probability, and prevalence. However, the remission patterns with time are similar for different cut-offs.

© 2025 Seitz-Holland J et al. *JAMA Network Open*.

eFigure 11: Incidence and Prevalence of Remission at Each Visit for Individuals That Did Not Use Antipsychotic Medication at Baseline.

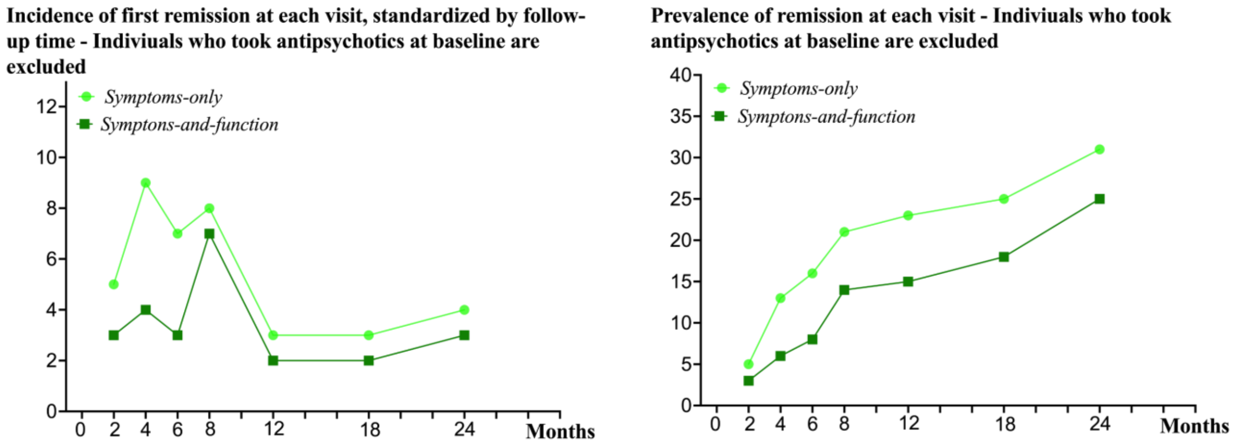

eFigure 10 shows the incidence and prevalence of remission for each follow-up visit. Individuals who already reported antipsychotic use at baseline were excluded from the analyses. Please note that the numbers are very similar to what has been reported for the whole sample (**Figure 1**).

© 2025 Seitz-Holland J et al. *JAMA Network Open*.

eTable 11: Comparison of Baseline Demographic and Clinical Data Between Groups Based on the Number of Follow-Up Visits.

Number of follow-up visits		0 (n=78)	1 (n=84)	2 (n=57)	3 (n=79)	4 (n=81)	5 (n=94)	6 (n=92)	7 (n=127)	Test statistic
Symptoms-and-function	Non-remitters: n (%)		78 (93%)	54 (95%)	72 (91%)	65 (80%)	68 (72%)	58 (63%)	89 (70%)	$X^2(14)$ = 745.8, p< .001 **
	Remitters: n (%)		6 (7%)	3 (5%)	7 (9%)	16 (20%)	26 (28%)	34 (37%)	38 (30%)	
Symptoms-only	Non-remitters: n (%)		73 (87%)	51 (89%)	61 (77%)	49 (60%)	55 (59%)	42 (46%)	72 (57%)	$X^2(14)$ = 759.4, p< .001 **
	Remitters: n (%)		11 (13%)	6 (11%)	18 (23%)	32 (40%)	39 (41%)	50 (54%)	55 (43%)	
	No follow-up	78								
Age (years)	Mean (std)	18.2 (4.4)	18.9 (3.8)	18.9 (3.8)	18.6 (3.8)	18.8 (4)	18.7 (4.4)	18 (4.2)	19.3 (3.8)	F(7) = 1, p=0.4
Sex	Female: n (%)	37 (47%)	33 (39%)	22 (39%)	41 (52%)	40 (49%)	46 (49%)	42 (46%)	58 (46%)	$X^2(7)$ = 4.7, p=0.7
Race	American Indian/ Alaska Native: n (%)	2 (3%)	1 (1%)			2 (2%)		4 (4%)	5 (4%)	$X^2(42)$ = 38.7, p=0.6
	Asian: n (%)	12 (15%)	12 (14%)	9 (16%)	4 (5%)	10 (12%)	10 (11%)	12 (13%)	12 (9%)	
	Black or African American: n (%)	7 (9%)	8 (10%)	9 (16%)	11 (14%)	9 (11%)	12 (13%)	7 (8%)	17 (13%)	
	Missing: n (%)					1 (1%)				
	More than one race: n (%)	9 (12%)	7 (8%)	8 (14%)	14 (18%)	10 (12%)	17 (18%)	10 (11%)	15 (12%)	
	Native Hawaiian or Pacific Islander: n (%)				1 (1%)				1 (1%)	

© 2025 Seitz-Holland J et al. *JAMA Network Open*.

	White: n (%)	48 (62%)	56 (67%)	31 (54%)	49 (62%)	49 (60%)	55 (59%)	59 (64%)	77 (61%)	
Antipsychotic use	n (%)	29 (37%)	30 (36%)	18 (32%)	29 (37%)	21 (26%)	38 (40%)	24 (26%)	27 (21%)	$X^2(7) = 15.0$, p=0.04 *
Antidepressant use	n (%)	44 (56%)	43 (51%)	28 (49%)	40 (51%)	40 (49%)	42 (45%)	36 (39%)	58 (46%)	$X^2(7) = 6.4$, p=0.5
Trauma	Trauma occurrence: n (%)	31 (40%)	42 (50%)	32 (56%)	42 (53%)	36 (44%)	41 (44%)	49 (53%)	66 (52%)	$X^2(7) = 6.8$, p=0.5
	Number of traumas: mean (std)	0.9 (1.2)	0.8 (0.9)	1.3 (1.3)	1.1 (1.2)	0.8 (1.1)	1.0 (1.2)	1.0 (1.2)	1.0 (1.2)	F(7) = 1.2, p=0.3
	Trauma impact: mean (std)	3.4 (5.2)	2.7 (3.4)	4.9 (5.7)	4.0 (5.0)	2.7 (4.2)	4.0 (5.4)	3.7 (4.6)	3.4 (4.4)	F(7) = 1.7, p=0.1
MATRICS: mean (std)	Hopkins Verbal Learning Test-Revised	26.4 (5)	26.4 (5.1)	25.2 (5.6)	26.6 (4.7)	25.7 (6)	25.8 (5.7)	26.5 (4.6)	27.4 (4.8)	F(7) = 1.6, p=0.1
	Brief Assessment of Cognition in Schizophrenia	51.9 (13.2)	55 (14.2)	53.6 (15.7)	54.9 (13.5)	54.4 (12.2)	54.3 (14.1)	53.6 (12.2)	55.8 (12.3)	F(7) = 0.7, p=0.7
	Letter-number-span	14.1 (3.9)	14.5 (4.9)	14.5 (4)	14.7 (3.8)	14.8 (3.6)	14 (3.7)	14.8 (3.6)	15 (2.8)	F(7) = 0.8, p=0.6
Wechsler Abbreviated Scale of Intelligence: mean (std)	Matrix score	20.4 (4.1)	20 (5.1)	20.8 (3.7)	21.8 (4.2)	21.1 (3.9)	20.3 (5.2)	21.3 (4.3)	21.2 (4.1)	F(7) = 1.5, p=0.2
	Vocabulary score	37 (7.4)	37 (8.3)	38 (6)	38.2 (6.3)	38.8 (5.7)	38.2 (6.7)	38.7 (6.8)	39.1 (5.3)	F(7) = 1.3, p=0.3
GAF: mean (std)	Current score	47.7 (10.5)	49.8 (10.9)	49.1 (12.5)	49.2 (12.5)	51.1 (12.7)	51.4 (13)	54.4 (11.4)	52.7 (11.7)	F(7) = 2.9, p=0.005 **

© 2025 Seitz-Holland J et al. *JAMA Network Open*.

	Highest score in the last year	56.6 (13.3)	55.1 (14.4)	57.6 (13.9)	56.2 (12.2)	58.8 (15.3)	58.2 (14.8)	58.9 (13.9)	60.1 (12.4)	F(7) = 1.3, p=0.2
SIPS: mean (std)	Positive	13.7 (3.5)	13.5 (3.7)	13.4 (3.2)	13.2 (3.6)	13.2 (3.2)	12.7 (3.5)	12.2 (3.4)	12.4 (2.8)	F(7) = 2.4, p=0.02**
	Negative	12.3 (6.7)	13.0 (6.6)	13.3 (6.9)	11.8 (5.8)	12.5 (5.3)	11.7 (6.6)	11.1 (6)	11.6 (6.5)	F(7) = 1, p=0.4
	Disorganized	5.8 (3.4)	5.8 (3.7)	5.2 (3.0)	4.9 (3.0)	5.2 (3.2)	4.8 (2.9)	4.8 (3.0)	5.1 (3.3)	F(7) = 1.4, p=0.2
	General	9.6 (4.4)	9.8 (3.9)	9.6 (4.4)	9.6 (4.3)	9.7 (3.8)	9.3 (4.7)	8.5 (4.4)	9.4 (4.1)	F(7) = 0.8, p=0.6

Abbreviations: GAF = Global Assessment of Functioning; SIPS = Structured Interview for Psychosis-risk Syndromes; std = standard deviation

Supplementary Table 4 is based on baseline data. Individuals were grouped based on how many follow-up visits they had. A conversion visit was counted as a follow-up visit. * Represents statistically significant group differences. ** Represents statistically significant group differences after correction for multiple comparisons using false discovery rate.

© 2025 Seitz-Holland J et al. *JAMA Network Open*.

eTable 12: Prevalence of Remission by Site at Each Visit.

Prevalence of remission at each visit – *symptoms-only*

	Site 1	Site 2	Site 3	Site 4	Site 5	Site 6	Site 7	Site 8	Site 9
Baseline	0/77, 0%	0/78, 0%	0/57, 0%	0/77, 0%	0/83, 0%	0/62, 0%	0/97, 0%	0/81, 0%	0/80, 0%
Month 2	3/39, 8% [0% -16.1%]	5/44, 11% [2.0% - 20.8%]	1/31, 3% [0% - 9.4%]	6/53, 11% [2.8% - 19.8%]	1/68, 2% [0% - 4.4%]	5/56, 9% [1.4% - 16.4%]	4/77, 5% [0.2% - 10.2%]	1/59, 2% [0% - 5.0%]	10/74, 14% [5.7% - 21.3%]
Month 4	3/38, 8% [0% - 16.5%]	8/42, 19% [7.1% - 30.9%]	4/24, 17% [1.8% - 31.6%]	9/50, 18% [7.4% - 28.6%]	6/61, 10% [2.3% - 17.3%]	9/54, 17% [6.8% - 26.6%]	11/72, 15% [7.0% - 23.6%]	2/58, 3% [0% - 8.1%]	9/63, 14% [5.7% - 22.9%]
Month 6	2/32, 6% [0% - 14.6%]	6/41, 15% [3.8% - 25.4%]	8/27, 30% [12.4% - 46.8%]	8/45, 18% [6.6% - 29.0%]	6/54, 11% [2.7% - 19.5%]	8/48, 17% [6.1% - 27.3%]	13/64, 20% [10.4% - 30.2%]	4/50, 8% [0.5% - 15.5%]	12/71, 17% [8.2% - 25.6%]
Month 8	2/35, 6% [0% - 13.4%]	8/43, 19% [7.0% - 30.2%]	5/28, 18% [3.7% - 32.1%]	12/47, 26% [13.0% - 38.0%]	10/57, 18% [7.6% - 27.4%	10/50, 20% [8.9% - 31.1%]	14/67, 21% [11.2% - 30.6%]	5/47, 11% [1.8% - 19.4%]	17/69, 25% [14.4% - 34.8%]
Month 12	3/23, 13% [0% - 26.7%]	8/41, 20% [7.4% - 31.6%]	8/27, 30% [12.4% - 46.8%]	12/50, 24% [12.2% - 35.8%]	12/56, 21% [10.7% - 32.1%]	11/48, 23% [11.0% - 34.8%]	15/64, 23% [13.0% - 33.8%]	8/45, 18% [6.6% - 29.0%]	20/65, 31% [19.6% - 42.0%]
Month 18	4/17, 24% [3.3% - 43.7%]	9/32, 28% [12.5% - 43.7%]	9/20, 45% [23.2% - 66.8%]	9/36, 25% [10.9% - 39.1%]	11/49, 22% [10.7% - 34.1%]	10/45, 22% [10.1% - 34.3%]	12/54, 22% [11.1% - 33.3%]	6/38, 16% [4.2% - 27.4%]	17/54, 32% [19.1% - 43.9%]
Month 24	8/28, 29% [11.9% - 45.3%]	11/31, 36% [18.7% - 52.3%]	8/20, 40% [18.5% - 61.5%]	13/36, 36% [20.4% - 51.8%]	9/40, 23% [9.6% - 35.4%]	8/35, 23% [9.0% - 36.8%]	14/45, 31% [17.6% - 44.6%]	7/35, 20% [6.7% - 33.3%]	14/41, 34% [19.6% - 48.6%]

Prevalence of remission at each visit – *symptoms-and-function*

	Site 1	Site 2	Site 3	Site 4	Site 5	Site 6	Site 7	Site 8	Site 9
Baseline	0/77, 0%	0/78, 0%	0/57, 0%	0/77, 0%	0/83, 0%	0/62, 0%	0/97, 0%	0/81, 0%	0/80, 0%
Month 2	3/39, 8% [0% - 16.1%]	3/44, 7% [0% - 14.2%]	0/31, 0%	1/53, 2% [0% - 5.6%]	0/68, 0%	3/56, 5% [0% - 11.3%]	2/77, 3% [0% - 6.2%]	0/59, 0%	6/74, 8% [1.9% - 14.3%]
Month 4	3/38, 8% [0% - 16.5%]	2/42, 5% [0% - 11.3%]	1/24, 4% [0% - 12.2%]	0/50, 0%	3/61, 5% [0% - 10.3%]	5/54, 9% [1.6% - 17.0%]	6/72, 8% [1.9% - 14.7%]	0/58, 0%	5/63, 8% [1.2% - 14.6%]
Month 6	1/32, 3% [0% - 9.1%]	2/41, 5% [0% - 11.5%]	1/27, 4% [0% - 10.8%]	1/45, 2% [0% - 6.5%]	4/54, 7% [0.4% - 14.4%]	6/48, 13% [3.1% - 21.9%]	6/64, 9% [2.3% - 16.5%]	1/50, 2% [0% - 5.9%]	8/71, 11% [3.9% - 18.7%]
Month 8	1/35, 3% [0% - 8.5%]	3/43, 7% [0% - 14.6%]	0/28, 0%	6/47, 13% [3.2% - 22.4%]	8/57, 14% [5.0% - 23.0%]	7/50, 14% [4.4% - 23.6%]	11/67, 16% [7.5% - 25.3%]	2/47, 4% [0% - 10.1%]	10/69, 15% [6.2% - 22.8]
Month 12	3/23, 13% [0% - 26.7%]	5/41, 12% [2.2% - 22.2%]	3/27, 11% [0% - 22.9%]	4/50, 8% [0.5% - 15.5%]	7/56, 13% [3.8% - 21.2%}	6/48, 13% [3.1% - 21.9%]	11/64, 17% [8.0% - 26.4%]	3/45, 7% [0% - 14.0%]	11/65, 17% [7.8% - 26.0%]
Month 18	3/17, 18% [0% - 35.7%]	6/32, 19% [5.3% - 32.3%]	4/20, 20% [2.5% - 37.5%]	6/36, 17% [4.5% - 28.9%]	4/49, 8% [0.5% - 15.9%]	8/45, 18% [6.6% - 29.0%]	8/54, 15% [5.3% - 24.3%]	4/38, 11% [0.8% - 20.2%]	11/54, 20% [9.7% - 31.1%]
Month 24	3/28, 11% [0% - 22.1%]	8/31, 26% [10.4% - 41.2%]	6/20, 30% [9.9% - 50.1%]	7/36, 19% [6.5% - 32.3%]	4/40, 10% [0.7% - 19.3%]	6/35, 17% [4.6% - 29.6%]	10/45, 22% [10.1% - 34.3%]	4/35, 11% [0.9% - 21.9%]	9/41, 22% [9.3% - 34.7%]

Individuals who converted at or before a visit were included in the analyses.

© 2025 Seitz-Holland J et al. *JAMA Network Open*.
