# Supplementary material for: Incidence, Prevalence, and Stability of Remission in Individuals With Clinical High Risk for Psychosis
Source: JAMA Netw Open. 2025 Aug 5;8(8):e2525644. doi: 10.1001/jamanetworkopen.2025.25644 (PMC12326281; doi:10.1001/jamanetworkopen.2025.25644)
Supplement: Supplement 2. — Data Sharing Statement [file jamanetwopen-e2525644-s002.pdf]

Data Sharing Statement

Seitz-Holland. Incidence, Prevalence, and Stability of Remission in Individuals With Clinical High Risk for Psychosis. *JAMA Netw Open*. Published August 05, 2025. doi:10.1001/jamanetworkopen.2025.25644

Data
Data available: Yes
Data types: Other (please specify)
Additional Information: The data is already publicly available. It was downloaded from NDA after a data use agreement.
How to access data: The data is already publicly available. It was downloaded from NDA after a data use agreement.
When available: With publication

Supporting Documents
Document types: None

Additional Information
Who can access the data: The data is already publicly available. It was downloaded from NDA after a data use agreement.
Types of analyses: The data is already publicly available. It was downloaded from NDA after a data use agreement.
Mechanisms of data availability: The data is already publicly available. It was downloaded from NDA after a data use agreement.
